# circCHST15 is a novel prognostic biomarker that promotes clear cell renal cell carcinoma cell proliferation and metastasis through the miR-125a-5p/EIF4EBP1 axis

**DOI:** 10.1186/s12943-021-01449-w

**Published:** 2021-12-18

**Authors:** Cheng-Peng Gui, Bing Liao, Cheng-Gong Luo, Yu-Hang Chen, Lei Tan, Yi-Ming Tang, Jia-Ying Li, Yi Hou, Hong-De Song, Hai-Shan Lin, Quan-Hui Xu, Gao-Sheng Yao, Hao-Hua Yao, Xi-Liu, Jun-Hang Luo, Jia-Zheng Cao, Jin-Huan Wei

**Affiliations:** 1 Department of Urology, First Affiliated Hospital, Sun Yat-sen University, Guangzhou, 510080 Guangdong China; 2 Institute of Precision Medicine, First Affiliated Hospital, Sun Yat-sen University, Guangzhou, Guangdong China; 3 Department of Pathology, First Affiliated Hospital, Sun Yat-sen University, Guangzhou, Guangdong China; 4 Department of Urology, Nanfang Hospital, Southern Medical University, Guangzhou, 510515 Guangdong China; 5 Department of Urology, Affiliated Jiangmen Hospital, Sun Yat-sen University, Jiangmen, 529000 Guangdong China

**Keywords:** hsa_circ_0020303, miR-125a-5p, EIF4EBP1, Proliferation, Metastasis, Clear cell renal cell carcinoma

## Abstract

**Background:**

Circular RNAs (circRNAs) have been indicated as potentially critical mediators in various types of tumor progression, generally acting as microRNA (miRNA) sponges to regulate downstream gene expression. However, the aberrant expression profile and dysfunction of circRNAs in human clear cell renal cell carcinoma (ccRCC) need to be further investigated. This study mined key prognostic circRNAs and elucidates the potential role and molecular mechanism of circRNAs in regulating the proliferation and metastasis of ccRCC.

**Methods:**

circCHST15 (hsa_circ_0020303) was identified by mining two circRNA microarrays from the Gene Expression Omnibus database and comparing matched tumor versus adjacent normal epithelial tissue pairs or matched primary versus metastatic tumor tissue pairs. These results were validated by quantitative real-time polymerase chain reaction and agarose gel electrophoresis. We demonstrated the biological effect of circCHST15 in ccRCC both in vitro and in vivo. To test the interaction between circCHST15 and miRNAs, we conducted a number of experiments, including RNA pull down assay, dual-luciferase reporter assay and fluorescence in situ hybridization.

**Results:**

The expression of circCHST15 was higher in ccRCC tissues compared to healthy adjacent kidney tissue and higher in RCC cell lines compared to normal kidney cell lines. The level of circCHST15 was positively correlated with aggressive clinicopathological characteristics, and circCHST15 served as an independent prognostic indicator for overall survival and progression-free survival in patients with ccRCC after surgical resection. Our in vivo and in vitro data indicate that circCHST15 promotes the proliferation, migration, and invasion of ccRCC cells. Mechanistically, we found that circCHST15 directly interacts with miR-125a-5p and acts as a microRNA sponge to regulate EIF4EBP1 expression.

**Conclusions:**

We found that sponging of miR-125a-5p to promote EIF4EBP1 expression is the underlying mechanism of hsa_circ_0020303-induced ccRCC progression. This prompts further investigation of circCHST15 as a potential prognostic biomarker and therapeutic target for ccRCC.

**Supplementary Information:**

The online version contains supplementary material available at 10.1186/s12943-021-01449-w.

## Introduction

Renal cell carcinoma (RCC) accounts for at least 3% of malignant diseases [1] and is the second leading cause of urological malignant neoplasm-related death [2]. Clear cell renal cell carcinoma (ccRCC) comprises 80–90% of RCC and has a higher invasive ability and a higher relapse rate than other RCC subtypes. In the past few decades, the incidence and mortality of ccRCC appear to be increasing rapidly [2]. Because of local recurrence and distant metastasis, overall patient survival is not satisfactory [3, 4]. Therefore, it is essential that the molecular mechanisms that promote ccRCC development and progression be further studied.

In the last decades, due to the development of high-throughput sequencing, numerous circRNAs have been discovered in mammalian cells [5–7]. And emerging evidence indicates that circRNAs may participate in the progression of many diseases, including cancers [8–15]. For instance, hsa_circ_001783 promoted progression of breast cancer cells via sponging miR-200c-3p [12]. circ-CPA4 regulated cell growth, mobility, stemness and drug resistance in NSCLC cells and inactivated CD8+ T cells in the tumor immune microenvironment through let-7 miRNA/PD-L1 axis [9]. And Hsa_circ_0058124 promotes papillary thyroid cancer tumorigenesis and invasiveness through the NOTCH3/GATAD2A axis [15]. circRNA hsa_circ_002577 accelerated EC progression by acting as a miR-625-5p sponge, upregulating IGF1R and activating the PI3K/Akt pathway [14].

Although multiple circRNAs have been reported to regulate tumor progression [16], the functional roles of circRNAs in ccRCC remain largely unknown. It has been shown that circTLK1 plays a critical role in RCC progression by sponging miR-136-5p, which increases CBX4 expression, and circTLK1 might act as a diagnostic biomarker and therapeutic target for RCC [10]. hsa_circ_001895 might sponge miR-296-5p and promote SOX12 expression, which is the underlying mechanism of hsa_circ_001895-induced ccRCC progression [8]. In this work, we discovered a novel circRNA, termed hsa_circ_0020303, which was upregulated in ccRCC specimens and associated with poor prognosis of patients with ccRCC. We determined that circCHST15 promotes proliferation and metastasis of ccRCC through directly binding to miR-125a-5p to attenuate miR-125a-5p-mediated suppression of EIF4EBP1. Taken together, our findings identify circCHST15 as a potentially novel prognostic biomarker and therapeutic target in ccRCC.

## Methods

### Microarray analysis

To identify differently expressed circRNAs in ccRCC, we obtained microarray expression data from the Gene Expression Omnibus (GEO) database (https://www.ncbi.nlm.nih.gov/geo/; GSE100186 and GSE137836). In addition, to verify the pan-cancer expression of circRNA, we obtained microarray expression data from the GEO database for bladder cancer (GSE147984), breast cancer (GSE165884), rectal cancer (GSE121895), gastric cancer (GSE141977), glioma (GSE146463), and lung cancer (GSE101684). We also analyzed data from The Cancer Genome Atlas (TCGA) cohort, which contained 527 patients from the TCGA-KIRC project, and the corresponding gene expression data were obtained from the Genomic Data Commons Data Portal (https://portal.gdc.cancer.gov).

Our study complied with the principles set forth in the Declaration of Helsinki, and access to the de-identified linked dataset was obtained from the TCGA and GEO databases in accordance with database policies. For analyses of de-identified data from the TCGA and GEO databases, institutional review board approval and informed consent were not required.

For all expression datasets from the GEO database, background correction and quartile normalization were performed for each series by applying the robust multi-array average algorithm [17]. The average value of gene symbols with multiple probes was calculated as expression level. For datasets from the TCGA database, messenger RNA (mRNA) expression was quantified with fragments per kilobase of exon per million reads mapped (FPKM). The primary prognosis endpoint was overall survival (OS), and survival curves were estimated using the Kaplan–Meier method. Expression profiles of circRNAs, miRNAs, and mRNAs were retrieved from two circRNA microarrays and the TCGA database. We applied the limma and edgeR packages to identify differentially expressed RNAs in ccRCC compared to matched adjacent epithelial tissue and in primary compared to matched metastatic tumor tissue. Next, a competing endogenous RNA (ceRNA) network was established based on circRNA–miRNA and miRNA–mRNA intersections. Significantly differentially expressed transcripts were defined as characterized by fold change ≥2 or ≤ − 2 and *P* ≤ 0.05.

### Patients and tumor tissue collection

We obtained 175 paired pathologically diagnosed ccRCC tissues and matched adjacent normal epithelial tissues from Sun Yat-sen University Cancer Center (Guangzhou, China). And all tissue specimens were immediately frozen in liquid nitrogen and stored at − 80 °C. The collection of tissue samples was approved by the Ethics Committee of Sun Yat-sen University Cancer Center. Moreover, we verified the identified circRNA expression levels in the Sun Yat-sen University (SYSU) patient cohort for prediction of OS and progression-free survival (PFS) by Kaplan–Meier analysis and compared them to clinicopathological characteristics and algorithms for clinical prognostic scores (stage, size, grade, necrosis [SSIGN] score).

### Cell culture and treatments

Human RCC cell lines 786-O, 769P, ACHN, CAKI-1, A498, and OSRC2, and the human renal proximal tubular epithelial cell line HK2 were purchased from the American Type Culture Collection. 786-O and 769P cells were cultured, in a humidified atmosphere of 5% CO2 maintained at 37 °C, in RPMI-1640 (Invitrogen-Gibco); CAKI-1 cells in McCoy’s 5A (Gibco); ACHN, OSRC2, HK2, and HEK293T cells in DMEM (Gibco); and A498 cells in MEM (Gibco). All cell culture media contained 10% fetal bovine serum (Thermo Fisher Scientific) and 1% penicillin-streptomycin (Gibco). When we performed the actinomycin D assay, we added 2 μg/ml of actinomycin D (Sigma-Aldrich) to 786-O and CAKI-1 cells, and incubated ccRCC cells for 4, 8, 16, and 24 h.

### RNase R treatment and quantitative real-time polymerase chain reaction

Genomic DNA and total RNA were extracted with MiniBEST Universal Genomic DNA Extraction Kit, version 5.0 (Takara) and RNAiso Plus (Takara), respectively. RNase R treatment was carried out for 30 min at 37 °C using 3 U/μg of RNase R (Epicenter Technologies). The nuclear and cytoplasmic fractions were isolated using a PARIS Kit (Life Technologies), according to the manufacturer’s instructions. RNA was reverse transcribed using PrimeScript RT Reagent Kit (Takara). TB Green Premix Ex Taq II (Takara) was used for qRT-PCR. The circRNA and mRNA levels were normalized by GAPDH. The miRNA level was normalized by small nuclear U6. Primers are listed in Additional file 1: Table S1.

### Oligonucleotide transfection

SiRNAs were synthesized by RiboBio (Guangzhou, China). Sequences used are listed in Additional file 2: Table S2. Transfection was carried out using RNAiMAX(Life Technologies) according to the manufacturer’s instructions.

### Plasmid construction and stable transfection

To construct circCHST15 over-expression plasmids, human circCHST15 cDNA was synthesized and cloned into a pLenti-CMV-GFP-Puro vector by GeneCreate (Wuhan, China), which was confirmed by sequencing. An empty plasmid served as the negative control. HEK-293 T cells were cotransfected with pLenti-CMV-GFP-Puro- circCHST15 or empty plasmid by Lipofectamine 2000 (Invitrogen). Forty-eight hours later, lentiviruses were harvested. ccRCC cells were infected with lentiviruses with 8 mg/mL polybrene by ViraPower Packaging Mix (ThermoFisher). Stable cell lines were obtained by treatment with 2 μg/mL puromycin (Sigma Aldrich) for 3 days. For silencing of circCHST15, sh-circCHST15 and sh-control were purchased from GenePharma.

### Cell proliferation, wound healing, migration, and invasion assays

For cell proliferation assay, 2 × 103 cells were seeded in 100 μl of complete culture media in 96-well plates for various time periods. Cell Counting Kit-8 assay (APExBio) was performed to measure cell viability according to manufacturer’s instructions.

For colony formation assays, 1 × 10^3^ cells were seeded in 6-well plates. Approximately 7–10 days later, the clones were then imaged and quantified.

For 5-ethynyl-2′-deoxyuridine (EdU) cell proliferation assays, we obtained the EdU kit from RiboBio to detect cell proliferation of 786-O, CAKI-1, HK2, or A498 cells. Cultured RCC cells (200 μl of 2 × 10^4^ cells/ml) were incubated with 50 μmol/L of EdU for 8 h. After fixation with 70% alcohol and permeabilization with Triton X-100, the cells were then incubated with Apollo staining reaction liquid (Click-iT EdU Apollo stain kit, Invitrogen) to label the cells. Nuclei were stained with DAPI. Immunostaining was visualized and photographed under a fluorescence microscope (Olympus inverted microscope IX71).

For wound healing assay, cells were seeded in 6-well plates with 5 × 10^5^ cells per well. Then, a wound was made by using a 200 μl pipette tip on the cell monolayer and photographs were taken at the appropriate time to estimate the area occupied by migratory cells.

For transwell invasion assays, a 24-well transwell chamber (Costar, USA) with Matrigel (BD Biosciences) was used to detect cell invasive ability according to the manufacturer’s protocol. Cells suspended in 0.2 ml serum-free medium (5 × 104/well) were added to the upper chambers, and media supplemented with 10% FBS was applied to the lower chambers. After incubating the cells for 6 h (for 786-O and HK2), 20 h (for CAKI-1) and 24 h (for A498), at 37 °C; and 5% CO2, cells that invaded to the lower membrane surface were fixed with 4% paraformaldehyde and stained with 1% crystal violet in PBS. Invaded cells were counted in five randomly selected fields. Three independent experiments were performed in triplicate.

### Western blot analysis

Proteins extracted from ccRCC tissues or cells (30 μg) were separated by SDS-PAGE, and then transferred to PVDF membrane. After blocking for 1 h with 5% skim milk powder at room temperature, membranes were incubated with primary antibodies specific to EIF4EBP1 (1:1000, Absin), vimentin (1:1000, Cell Signaling Technology), N-cadherin (1:1000, Cell Signaling Technology), E-cadherin (1:1000, Cell Signaling Technology), PCNA (1:1000, Cell Signaling Technology), p-AKT (1:1000, Cell Signaling Technology), AKT (1:1000, Cell Signaling Technology), mTOR (1:1000, Cell Signaling Technology), p-mTOR (1:1000, Cell Signaling Technology), PI3K (1:1000, Cell Signaling Technology), p-PI3K (1:1000, Cell Signaling Technology), or GAPDH (1:5000, Abcam) at 4 °C overnight. The membranes were then incubated with horseradish peroxidase (HRP)-conjugated goat anti-mouse secondary antibody (1:5000, Cell Signaling Technology) and visualized using the Immobilon Western Chemiluminescent HRP Substrate (Millipore).

### Biotin-coupled probe pull-down assay

The biotinylated probe was specifically designed to bind to the junction area of circCHST15, while the oligo probe was taken as a control. Approximately 1 × 10^7^ cells were harvested and lysed. The circCHST15 probe (GenePharma, Suzhou, China) was incubated with streptavidin magnetic beads (Life Technologies, USA) at room temperature for 2 h to generate probe-coated beads. The cell lysates were incubated with probe-coated beads at 4 °C overnight. The beads were washed and the bound miRNAs in the pull-down materials were extracted using Trizol reagent and analyzed by qRT-PCR assay.

### Biotin-coupled miRNA capture

The 3′ end biotinylated miRNA mimics or control RNA (Ribio, Guangzhou, China) were transfected into ccRCC cells for 48 h before harvest. Then biotin-coupled RNA complex was pulled down by incubating the cell lysates with streptavidin-coated magnetic beads (Life Technologies). The abundance of circCHST15 in bound fraction was evaluated by qRT-PCR analysis.

### Fluorescence in situ hybridization

The ccRCC cells were first fixed in 4% formaldehyde solution, and then incubated with 0.5% Triton X-100. The Cy5-labeled circCHST15 probe and Cy3-labeled miR-125a-5p probe (GenePharma, Suzhou, China) were hybridized at 37 °C with cells in the dark for 5 h. The cells were then photographed by laser scanning confocal microscopy (Carl Zeiss). The sequences of the probes are listed in Additional file 3: Table S3.

### Luciferase reporter assay

Wild-type or mutant circCHST15 or 3′-UTR EIF4EBP1 was synthesized and then subcloned into psiCHECK-2 vector. HEK293T cells were seeded in 24-well plates at a concentration of 1.5 × 105 per well and cotransfected miR-125a-5p mimics or miR-NC with psiCHECK-2-wt-circCHST15, psiCHECK-2-mut-circCHST15,

psiCHECK-2-wt-EIF4EBP1 or psiCHECK-2-mut-EIF4EBP1. Two days later, luciferase activities were measured by Lucifer Reporter Assay System (Promega) and normalized to Renilla luciferase activity.

### Hematoxylin and eosin staining and immunohistochemical analysis

The primary antibodies specific for EIF4EBP1(Absin), vimentin (Cell Signaling Technology), N-cadherin (Cell Signaling Technology), and E-cadherin (Cell Signaling Technology) were used at the appropriate dilution in the experiments. Tissue samples of 5-μm-thick paraffin sections were stained with hematoxylin and eosin and subjected to immunohistochemical analysis. Images were captured using a Nikon Eclipse 80i system with NIS-Elements software.

### Animal experiments

BALB/c nude mice (4–6 weeks old) were purchased from Charles River Laboratories. All animal care and experimental procedures were approved by the Institutional Animal Care and Use Committee of Sun Yat-sen University and were performed in accordance with established guidelines.

For the tumor growth study, eight mice were included in each group, and 786-O cells with stable knockdown of circCHST15 or control cells (5 × 10^6^ cells per mouse) were injected subcutaneously into the left side of the axilla. The size of the tumor was measured every week. Four weeks later, the mice were sacrificed, and tumor weight was recorded.

An in vivo metastasis model was established by intravenous injection through the tail vein of 2 × 10^6^ ccRCC cells that stably expressed firefly luciferase into 4-week-old BALB/c nude mice. Six weeks after injection, bioluminescence of lung metastases of tumor was detected using an in vivo bioluminescence imaging system. Then, the mice were sacrificed. The lungs were removed and fixed with phosphate-buffered formalin. Subsequently, consecutive tissue sections were made for each block of the lung. The numbers of pulmonary metastatic nodules in the lung were recorded.

### Statistical analysis

Statistical analyses were conducted using SPSS 19.0 or GraphPad Prism 8.0 software. Student’s *t*-test (two tailed) was applied to assess the statistical significance between two groups. The chi-square test was used to analyze the correlation between circCHST15 expression levels and clinicopathological features in ccRCC. OS curves were calculated with the Kaplan–Meier method and analyzed with the log-rank test. Data were presented as the mean ± standard error of the mean. *P* < 0.05 was considered statistically significant.

## Results

### Key circRNAs were identified by microarray data analysis

**Fig. 1:**
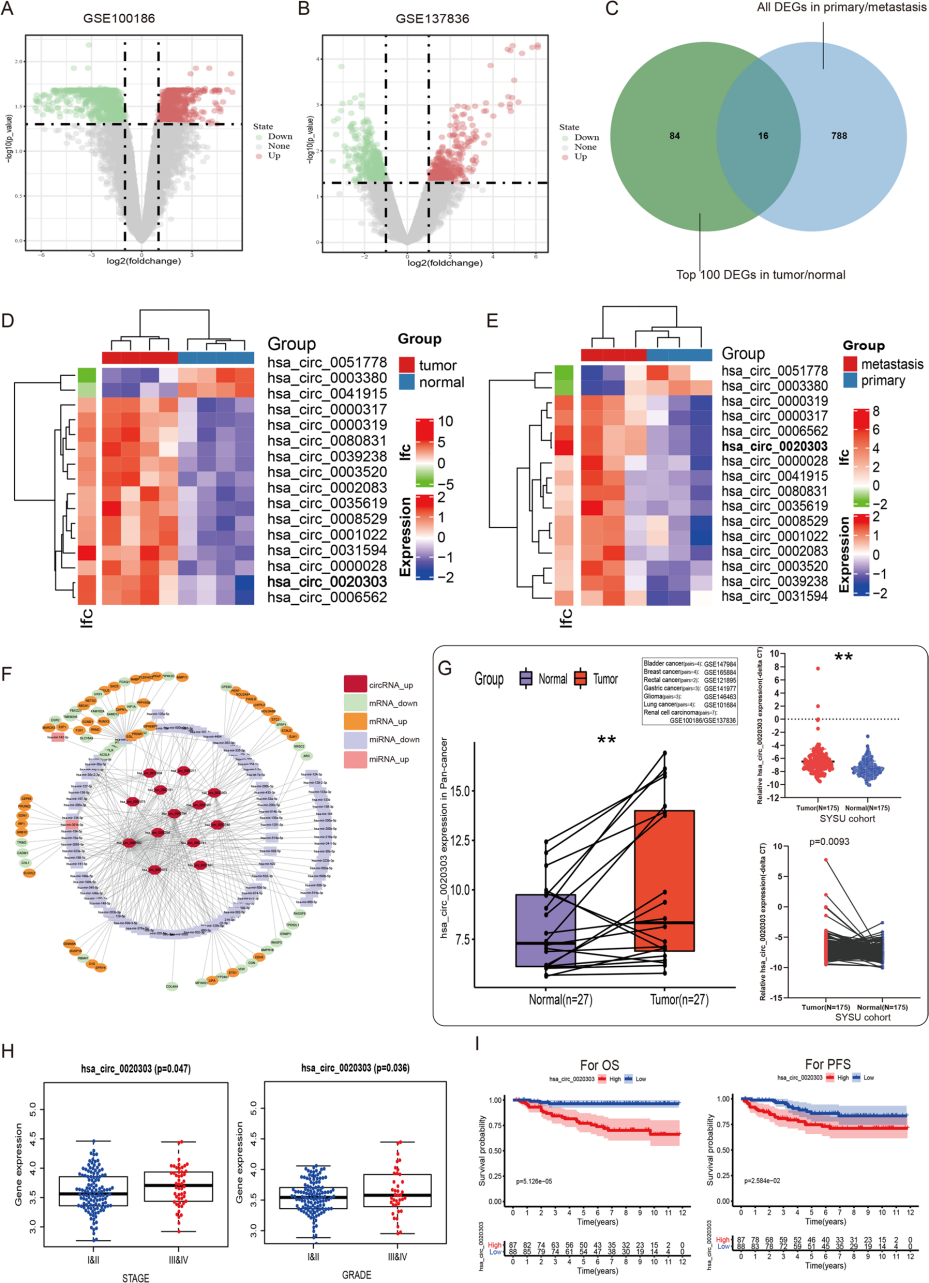
Identification of has_circ_0020303 in ccRCC tissues. **A** Volcano plot of GSE137836 and GSE100186. Compared to the primary tumors (in GSE137836) and adjacent normal tissue (in GSE100186), red dots represent significantly up-regulated circRNAs, while green dots represent significantly down-regulated circRNAs. Grey dots represent circRNAs that are not significant. **B** Venn plot of the two datasets. Common circRNAs with *p* < 0.05, |log2 (fold change)| > 1 are chosen. **C** identification of sixteen circRNAs from the overlaps of top 100 DEGs in two circRNA microarrays. **D** Heat map shows dysregulated circRNAs between ccRCC tissues (T) and adjacent noncancer tissues (N). The numerical data represented the serial number of circRNAs in circBase. **E** Heat map shows dysregulated circRNAs between metastatic ccRCC tissues and primary ccRCC tissues. **F** a ceRNA network was established based on circRNA-miRNA and miRNA-mRNA intersections. **G** Expression of circRNA hsa_circ_0020303 in pan-cancer analysis or in ccRCC tissues and adjacent noncancer tissues detected by qRT-PCR (*N* = 175). **H** Correlation between circRNA hsa_circ_0020303 and clinical characteristics. **I** Overall survival and progression-free survival analysis of ccRCC patients with high hsa_circ_0020303 expression and low levels of hsa_circ_0020303

We simultaneously analyzed the expression profiles of circRNAs in two microarray datasets, which contained four matched tumor/normal adjacent epithelial tissue sample pairs and three matched primary/metastatic tumor tissue pairs (GSE100186 and GSE137836; www.ncbi.nlm.nih.gov/geo). A total of 673 dysregulated circRNAs were identified in GSE100186, and 804 were identified in GSE137836. Then, 16 circRNAs were prioritized from among the top 100 circRNAs that were differently expressed in both of the GEO datasets (Fig. 1A–E). Then, we queried the expression of these 16 circRNAs across multiple cancer types, which identified circCHST15 as a probable oncogene (Fig. 1G). Additionally, according to the method of bioinformatics analysis, we predicted the possible circRNA–miRNA and miRNA–mRNA intersections, and drew a ceRNA network map by using the Software Cytoscape software (Fig. 1F).

### circCHST15 is upregulated in ccRCC and associated with poor prognosis of patients with ccRCC

To validate the clinical relevance of circCHST15 in ccRCC, we first applied qRT–PCR to detect the expression of circCHST15 in 175 pairs of ccRCC tissues and matched adjacent normal tissues. It was identified that circCHST15 was significantly upregulated in ccRCC tissue than adjacent normal tissue (Fig. 1G). Subsequently, it was analysed that the over-expression of circCHST15 was positively correlated with advanced tumor stage, while not significantly correlated with other clinicopathological features (Table 1). We also determined that more advanced tumor grade and stage was associated with higher circCHST15 expression level, and Kaplan–Meier analysis showed that patients with tumors characterized by low circCHST15 expression had markedly better OS and PFS (Fig. 1H,I). In addition, receiver operating characteristic (ROC) analysis found that circCHST15 level can be considered in combination with other clinical indicators to more accurately predict patient OS, such as ROC_SSIGN + circCHST15_ = 0.887, ROC_SSIGN_ = 0.844, ROC_circCHST15_ = 0.718, and *P* (for ROC test) < 0.011 (Fig. S2A,C). circCHST15 can also improve the PFS predictive ability of the SSIGN scoring scale (Fig. S2B,D). No relationship between age or gender and circCHST15 level was observed (Fig. 1H).

**Table 1:** Correlations between CircCHST15(hsa_circ_0020303) expression levels and clinicopathological characteristics in ccRCC(SYSU cohort, *n* = 175)

Characteristics	cases	CircCHST15 expression		*P*-value
		Low	High	
Age(years)				0.3242
< 60	123	65	58	
≥ 60	52	23	29	
Gender				ns
Male	121	61	60	
Female	54	27	27	
AJCC stage_T				0.0009*
T1/T2	137	78	59	
T3/T4	38	10	28	
AJCC stage_N				0.9886
N0	169	85	84	
N1	6	3	3	
AJCC stage_M				0.0414*
M0	162	85	77	
M1	13	3	10	
STAGE				0.0064*
I/II	125	71	54	
III/IV	50	17	33	
GRADE				0.1532
I/II	135	72	63	
III/IV	40	16	24	

*P < 0.05 was considered to be statistically significant (chi-square test)

*AJCC* American Joint Committee on Cancer; SYSU cohort, 175 patients with ccRCC from Sun yat-sen University Cancer Center

In addition, we conducted univariate Cox regression analysis in the SYSU cohort to determine whether circCHST15 expression level and clinical features (including age, sex, T stage, N stage, M stage, stage, tumor grade SSIGN score) might be valuable prognostic biomarkers. Univariate analysis uncovered a significant, positive correlation between increasing circCHST15 expression level and clinical features (except sex) for both OS and PFS (Tables 2 and 3). To ascertain whether circCHST15 expression level could be an independent prognostic factor for patients with ccRCC, multivariate Cox regression analysis was performed. We verified that increased circCHST15 expression level was a significant independent prognostic factor in the SYSU cohort that directly correlated with poorer OS and PFS outcomes (Tables 2 and 3).

**Table 2:** Univariate and multivariate Cox regression analyses of different parameters on overall survival

parameter	Univariate Analysis		Multivariate Analysis	
	HR(95%CI)	*P* Value	HR(95%CI)	*P* Value
Age(≥60 yr vs. < 60 yr)	2.887(1.336–6.239)	0.007	2.781(1.206–6.412)	0.016
Sex(male vs. female)	2.303(0.867–6.116)	0.094	2.665(0.952–7.456)	0.062
Stage(III/IV vs. I/II)	10.416(4.349–24.947)	< 0.001	5.444(2.133–13.894)	< 0.001
Grade(3/4 vs.1/2)	5.259(2.415–11.451)	< 0.001	2.819(1.204–6.601)	0.017
circCHST15 expression level (High vs. Low)	8.234(2.472–27.429)	< 0.001	8.948(2.620–30.554)	< 0.001

*HR* hazard radio, *CI* confidence interval

**Table 3:** Univariate and multivariate Cox regression analyses of different parameters on progression-free survival

parameter	Univariate Analysis		Multivariate Analysis	
	HR(95%CI)	*P* Value	HR(95%CI)	*P* Value
Age(≥60 yr vs. < 60 yr)	2.381(1.198–4.733)	0.013	2.028(0.985–4.174)	0.055
Sex(male vs. female)	1.997(0.866–4.606)	0.105	2.276(0.966–5.367)	0.060
Stage(III/IV vs. I/II)	4.617(2.309–9.234)	< 0.001	2.480(1.123–5.477)	0.025
Grade(3/4 vs.1/2)	4.201(2.102–8.397)	< 0.001	2.899(1.323–6.351)	0.008
circCHST15 expression level (High vs. Low)	2.282(1.106–4.707)	0.026	2.619(1.244–5.512)	0.011

*HR* hazard radio, *CI* confidence interval

### Identification and characterization of circCHST15 in ccRCC cells

**Fig. 2:**
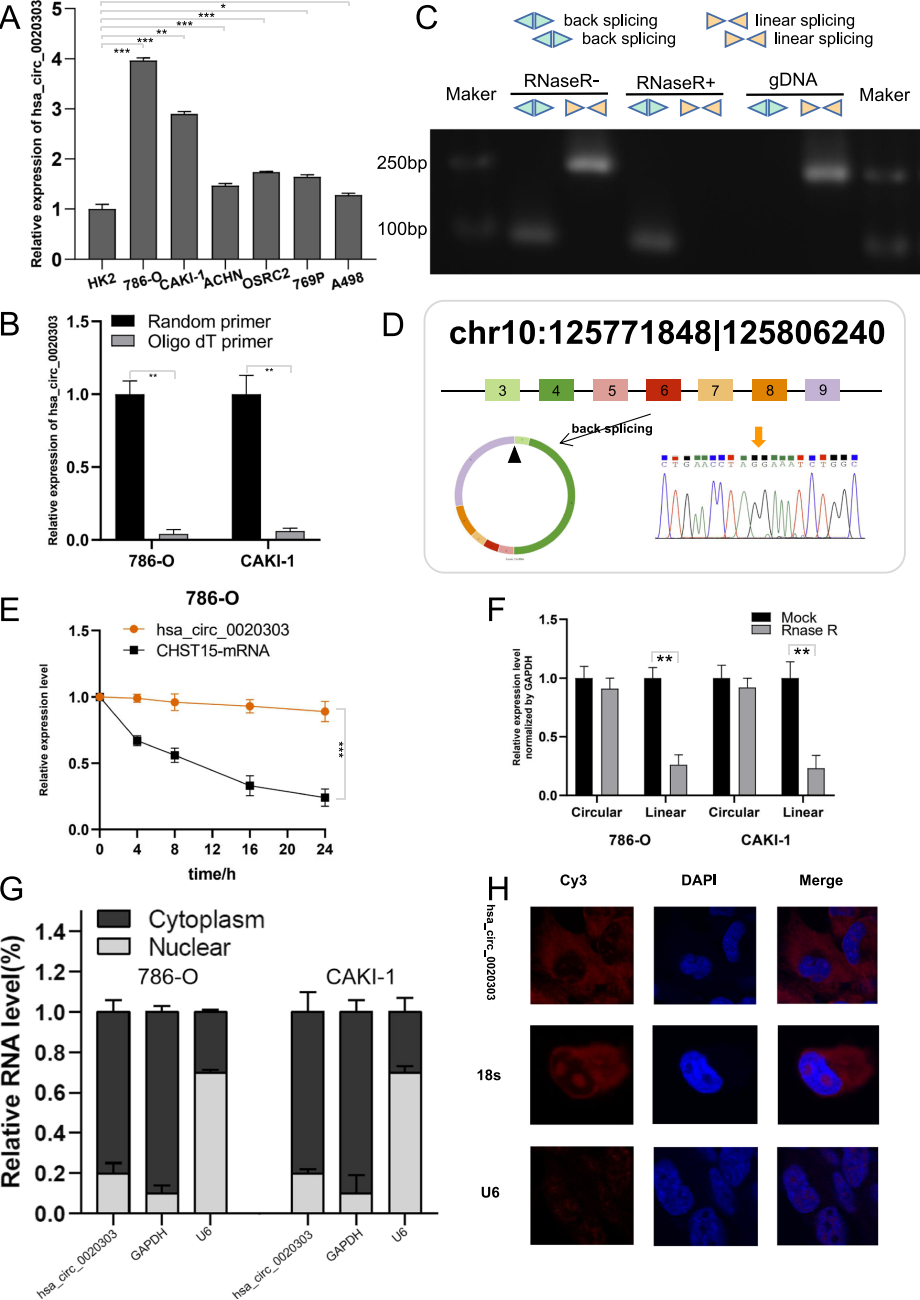
The validation and characteristics of circCHST15 in RCC cells. **A** Relative expression of circCHST15 in the human renal proximal tubular epithelial cell line HK2immortalized uroepithelium cell line HK2, and RCC cell lines 786-O, 769P, ACHN, CAKI-1, A498 and OSRC2. **B** qRT-PCR analysis of circCHST15 in the reverse transcription products using random primers or oligo dT primers. **C** The existence of circ CHST15 was detected in 786-O cell lines by qRT-PCR with convergent or divergent primers and validated by Gel electrophoresis. **D** The expression of circCHST15 was validated by Sanger sequencing. Yellow arrow represents the back-splicing site of circCHST15. CircCHST15 derived from back-splicing of exons 2,3, 4,5,6,7,8 and 9 of CHST15 gene. **E** qRT-PCR analysis of circ CHST15 and CHST15 mRNA after treatment with Actinomycin D at the indicated time points in 786-O cells. **F** qRT-PCR analysis of circ CHST15 and CHST15 mRNA after treatment with or without RNase R in 786-O and CAKI-1 cells. **G** qRT-PCR analysis of circCHST15 using nuclear and cytoplasmic fractions of 786-O and CAKI-1 cells. **H** FISH confirmed that circCHST15 was predominantly located in cytoplasm. Nuclei were stained with DAPI. U6, 18S and circCHST15 were labeled with Cy3. **K-L** Influence of hsa_circ_0020303 on cell proliferation of ccRCC cells detected by 5-ethynyl-2′-deoxyuridine (EdU) staining assay

As mentioned above, we first analyzed the published circRNA microarray data of human ccRCC tissues and paired normal renal tissues and found that circCHST15 was increased in ccRCC. Interestingly, based on our ceRNA network predictive model, circCHST15 might also function as a miRNA sponge. To test this hypothesis, we first confirmed that the expression of circCHST15 was significantly upregulated in 786-O, 769P, ACHN, CAKI-1, A498, and OSRC2 RCC cell lines compared to the human renal proximal tubular epithelial cell line HK2 (Fig. 2A). Because circRNAs do not have a 3′ polyadenylated tail, we detected the presence of circCHST15 in the reverse transcription products using random primers or oligo dT primers, and we verified that circCHST15 was almost undetectable when oligo-dT primers were used (Fig. 2B). To verify that exons 3, 4, 5, 6, 7, 8, and 9 of the *CHST15* gene formed an endogenous circRNA, we designed divergent and convergent primers that specifically amplified the back-spliced and canonical forms of *CHST15* (Fig. 2C,Fig. S3A). circCHST15 was detected by RT–PCR with divergent primers, and the amplified fragment was resistant to digestion by RNase R. In contrast, convergent primers amplified the CHST15 mRNA, which disappeared after RNase R digestion. Furthermore, no genomic DNA was amplified using the divergent primers, eliminating artifacts caused by genomic rearrangement (Fig. 2C, Fig. S3B).

Subsequently, Sanger sequencing of the qRT–PCR product of circCHST15 was performed to verify the head-to-tail splicing. The result was in accordance with circBase (http://circrna.org/), which indicated that circCHST15 was derived from exons 3, 4, 5, 6, 7, 8, and 9 of the *CHST15* gene (Fig. 2D).

Next, the actinomycin D assay showed that the half-life of circCHST15 transcript exceeded 24 h, indicating that this isoform is more stable than the linear CHST15 mRNA transcript in 786-O cells (Fig. 2E). Consistently, the RNase R treatment showed that circCHST15 was resistant to RNase R (Fig. 2F). Next, it was clearly identified that circCHST15 was enriched in the cytoplasm through sub-cellular localization analysis and FISH assay (Fig. 2G,H).

### circCHST15 promotes proliferation, migration, and invasion of ccRCC cells in vitro

**Fig. 3:**
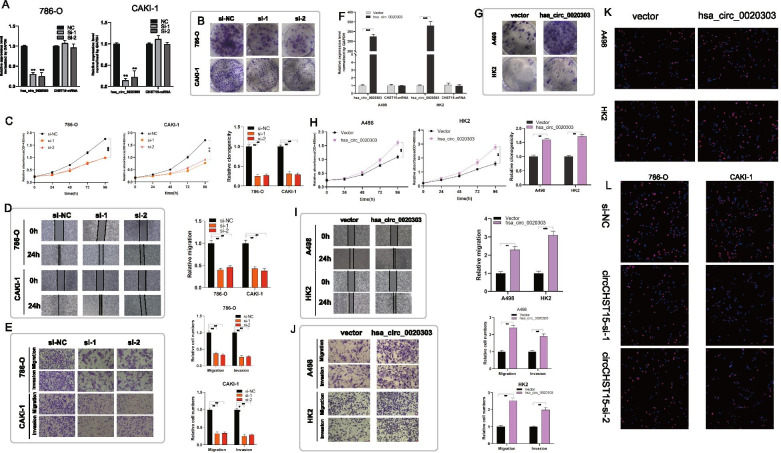
Knockdown/overexpression of circCHST15 suppresses/promote RCC cells proliferation, migration and invasion in vitro. **A-E** 786-O and CAKI-1 cells after transfection of circCHST15-siRNA or siRNA-control. **F-J**. A498 and HK2 cells after stable transfection of circCHST15 or vector. **A**, **F** qRT-PCR analysis of circCHST15 and CHST15 mRNA in RCC cells and HK2 cells. **B-C** G-H Cell proliferation ability of RCC cells and HK2 cells was evaluated by colony formation assay and CCK8 assay. **D**, **I** Cell migration capability of RCC cells and HK2 cells was evaluated by wound healing assays. **E-J** The influence on cell migration and invasion abilities of RCC cells and HK2 cells was assessed by transwell migration and matrigel invasion assays

To discover the potential biological effect of circCHST15 in ccRCC cells, we used RNA interference (RNAi) to silence the expression of circCHST15 in 786-O and CAKI-1 cells, and we also engineered these cell lines to stably overexpress circCHST15 via transfection with a circCHST15 vector. The knockdown and overexpression efficiencies in transformed cell lines were detected by qRT–PCR analysis (Fig. 3A,F). Importantly, the expression level of genomic *CHST15* was not affected by circCHST15 changes (Fig. 3A,F). It was confirmed that knockdown of circCHST15 reduced the proliferative capacity of 786-O and CAKI-1 cells (Fig. 3B,C,L) upon colony formation assays, CCK8 assays, and EdU assays. Moreover, it was revealed that down-regulation of circCHST15 suppressed cell migration in 786-O and CAKI-1 cells. Additionally, transwell assays revealed that the migration and invasion abilities of ccRCC cells were significantly inhibited through down-regulating circCHST15 expression level (Fig. 3D,E). On the contrary, it was proved that up-regulation of circCHST15 promoted the proliferation, migration, and invasion of ccRCC cells (Fig. 3G–K). Briefly, it was suggested that circCHST15 plays a role of oncogene in ccRCC cells.

### circCHST15 serves as a miRNA sponge for miR-125a-5p in ccRCC cells

**Fig. 4:**
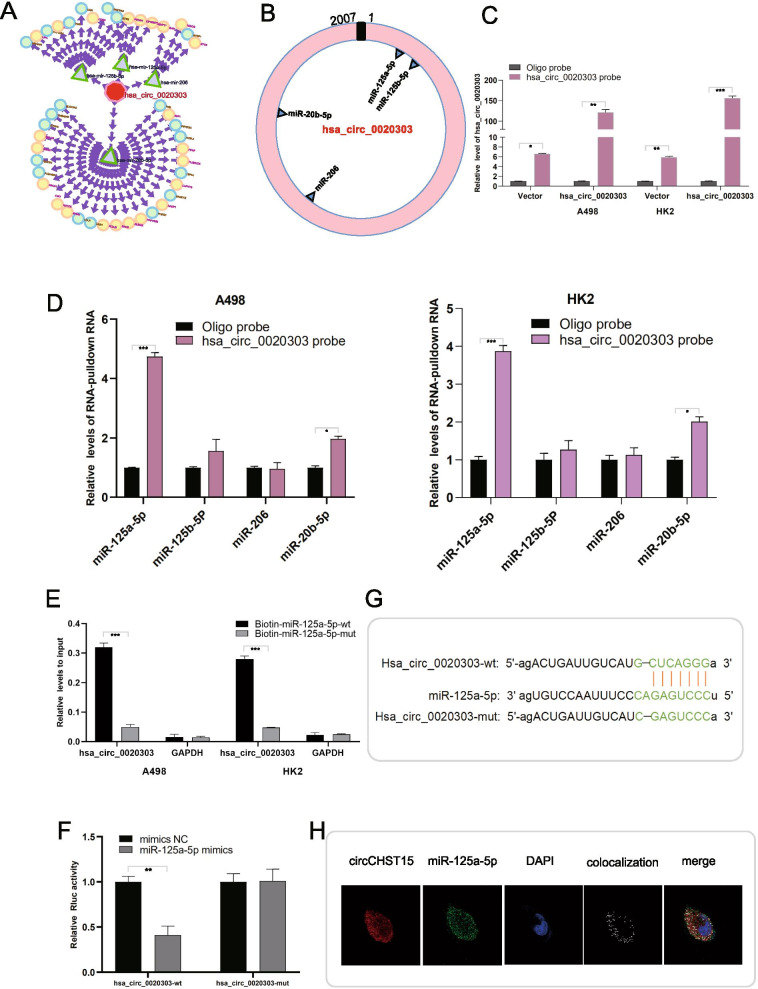
circCHST15 serves as a miR-125a-5p sponge in RCC cells. **A** Four potential target miRNAs of circCHST15 were predicted by bioinformatic analysis. **B** Schematic model showed the putative binding sites of eight miRNA candidates associated with circCHST15. **C**. Relative levels of circCHST15 in A498 and HK2 lysates after RNA pull down using circCHST15 probe or oligo probe. **D** Relative levels of four miRNAs in A498 and HK2 lysates pulled down by circCHST15 probe or oligo probe. **E** Relative levels of circCHST15 in A498 and HK2 lysates captured by biotinylated wild-type miRNA-125a-5p (Biotin-miR-125a-5p-wt) or mutant miR-125a-5p(Biotin-miR-125a-5p-mut). **F** Luciferase reporter assay in HEK293T cells co-transfected with miRNA mimics, psiCHECK-2-wild type circCHST15 (circCHST15-wt) or psiCHECk-2-mutant type circCHST15 (circCHST15-mut) plasmids. **G** Schematic of circCHST15 wild-type (wt) and mutant (mut) luciferase reporter vectors. **H** FISH showed the colocalization between circCHST15 and miR-125a-5p in A498 cells. Nuclei were stained with DAPI. CircCHST15 was labeled with Cy3 and miR-125a-5p was labeled with Cy5

Because circRNAs predominantly located in the cytoplasm are usually associated with miRNA sponging [18, 19], we further explored whether circCHST15 could bind to miRNAs. Four potential target miRNAs (miR-206, miR-20b-5p, miR-125a-5p, and miR-125b-5p) were predicted by CircInteractome and miRanda to interact with circCHST15, and these miRNAs were selected as candidate miRNAs for subsequent experiments (Fig. 4A). The positions of putative binding sites in circCHST15 are shown in Fig. 4B. We designed a biotinylated circCHST15 probe and oligo probe for RNA pull-down assays, and we verified the pull-down efficiency of the probes in A498 and HK2 cells transfected with circCHST15 overexpression or control vector (Fig. 4C). The miRNAs pulled down by biotinylated probes were purified and analyzed by qRT–PCR. Of the four candidate miRNAs, only miR-125a-5p could be abundantly pulled down by circCHST15 probe in both A498 and HK2 cells overexpressing circCHST15 (Fig. 4D). To further confirm the sponging of miR-125a-5p by circCHST15, we conducted biotin-coupled miRNA capture and luciferase reporter assays, as well as FISH. A498 and HK2 cells stably overexpressing circCHST15 were transfected with biotin-labeled miR-125a-5p (biotin-miR-125a-5p-wt) or its mutant (biotin-miR-125a-5p-mut), and circCHST15 captured by miR-125a-5p was evaluated by qRT–PCR. We detected robust enrichment of circCHST15 in the captured fraction from biotin-miR-125a-5p-wt cells compared to biotin-miR-125a-5p-mut cells (Fig. 4E), supporting that miR-125a-5p can bind to circCHST15. As an orthogonal approach, we inserted either wild-type circCHST15 sequence or circCHST15 sequence with mutations in the miR-125a-5p binding sites into the psiCHECK-2 vector to perform luciferase reporter assays. HEK293T cells were transfected with miRNA mimics and either wild-type or mutant circCHST15 psiCHECK-2 vector for 48 h, and then Renilla luciferase activity was quantified. When we up-regulated the miR-125a-5p levels, relative luciferase activity was decreased. It was suggested that miR-125a-5p interacts with circCHST15 (Fig. 4F,G). Besides, it was showed that circCHST15 and miR-125a-5p were co-localized in the cytoplasm (Fig. 4H) by FISH analysis. Collectively, it was suggested that circCHST15 might act as a ceRNA through targeting miR-125a-5p.

### miR-125a-5p is downregulated and exerts a tumor-suppressive role by targeting EIF4EBP1 in ccRCC cells

**Fig. 5:**
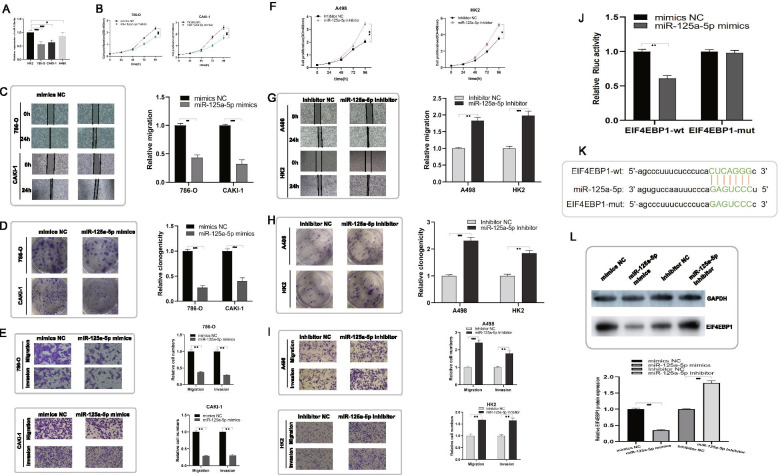
Up-regulated/down-regulated miR-125a-5p suppresses/promotes cell proliferation, migration and invasion through targeting EIF4EBP1 in vitro. **A** Relative expression of miRNA-125a-5p in HK2, 786-O, CAKI1 and A498 cell lines. **B-E** 786-O and CAKI-1 cells transfected with mimics NC or miR-125a-5p mimics. **F-I**, A498 and HK2 cells transfected with Inhibitor NC or miR-125a-5p Inhibitor. **B**, **F** Cell proliferation ability of RCC cells and HK2 cells was evaluated by CCK8 assay. **C**, **G** Cell migration capability of RCC cells and HK2 cells was evaluated by wound healing assays. **D**, **H** Cell proliferation ability of RCC cells and HK2 cells was evaluated by colony formation assay. **E**, **I** The influence on cell migration and invasion abilities of RCC cells and HK2 cells was assessed by transwell migration and matrigel invasion assays. **J** Luciferase reporter assay in HEK293T cells co-transfected with miRNA mimics, psiCHECK-2-wild type EIF4EBP1 (EIF4EBP1-wt) or psiCHECk-2-mutant type EIF4EBP1 (EIF4EBP1-mut) plasmids. **K** Schematic of EIF4EBP1 wild-type (wt) and mutant (mut) luciferase reporter vectors. **L** Western blot analysis indicated that miR-125a-5p could down-regulate EIF4EBP1 expression in RCC cells; histogram for protein expressions among different groups. *, *p* < 0.05; **, *p* < 0.01; ***, *p* < 0.001

Because of the clinch between circCHST15 and miR-125a-5p, we assessed the expression level and potential effect of miR-125a-5p in ccRCC cells. It was analysed that miR-125a-5p was notably suppressed in ccRCC cell lines compared to HK2 cells (Fig. 5A) upon RT–PCR assay. To evaluate the functional role of miR-125a-5p, we transfected miRNA mimics or control constructs in 786-O, CAKI-1, Cell proliferation, migration, and invasion abilities were significantly suppressed in ccRCC cells transfected with miR-125a-5p mimics compared to the control group (Fig. 5B–E). We also transfected A498 and HK2 cells with either a miRNA inhibitor or a control construct. In contrast to what we observed with the miRNA mimics, inhibiting miR-125a-5p significantly promoted the proliferation, migration, and invasion capabilities of ccRCC cells (Fig. 5F–I).

To identify possible target genes of miR-125a-5p in ccRCC cells, we applied miRDB [20] and TargetScan [21]. Accordingly, it was revealed that EIF4EBP1 might act as a potential target gene of miR-125a-5p. And then we conducted luciferase reporter assays. The results showed that miR-125a-5p mimics decreased the luciferase activity of psiCHECK-2-wt-EIF4EBP1, but had no effect on luciferase activity of psiCHECK-2-mut-EIF4EBP1 (Fig. 5J,K). Consistently, it was indicated that the expression of EIF4EBP1 protein was significantly reduced after transfection of miR-125a-5p mimics, while upregulated after transfection of miR-125a-5p inhibitor in A498 and HK2 cells by western blot analysis (Fig. 5L).

**Fig. 6:**
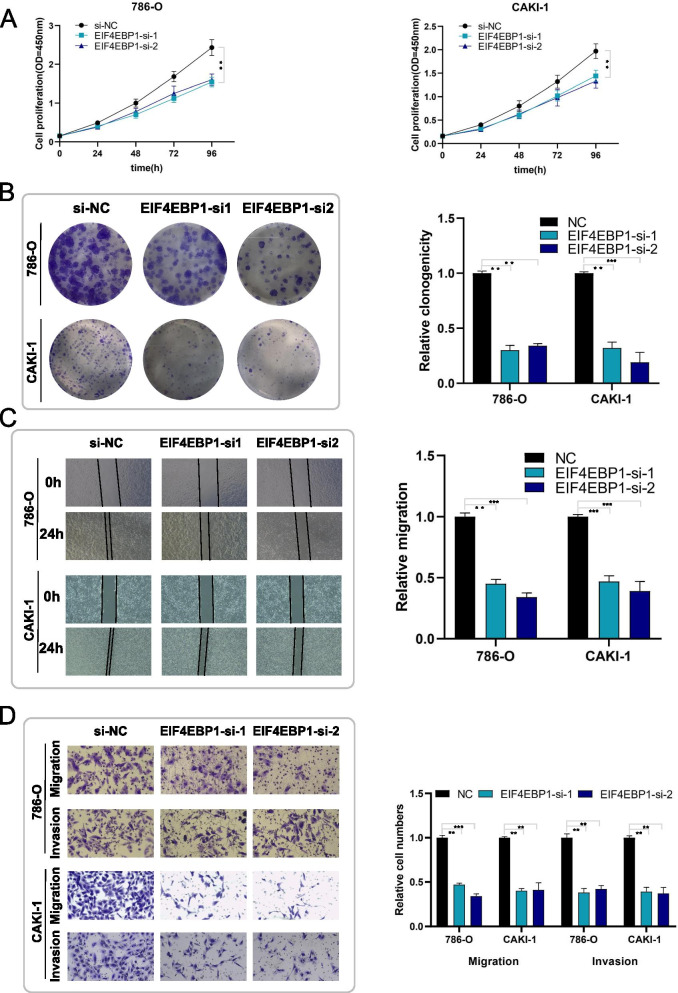
Down-regulation of EIF4EBP1 suppresses proliferation, migration and invasion of RCC cells in vitro. **A-B** Cell proliferation ability of 786-O and CAKI-1 cells transfected with EIF4EBP1 siRNAs was evaluated by CCK8 assay and colony formation assay. **C** Cell migration capability of 786-O and CAKI-1 cells transfected with EIF4EBP1 siRNAs was assessed by wound healing assays. **D** The influence on cell migration and invasion abilities of 786-O and CAKI-1 cells transfected with EIF4EBP1 siRNAs was evaluated by transwell migration and matrigel invasion assay, respectively

Previous studies showed that *EIF4EBP1* acts as an oncogene in lung adenocarcinoma [22], hepatocellular carcinoma [23], and nasopharyngeal carcinoma [24], but no one has studied the biological effect of EIF4EBP1 in ccRCC. Our study revealed that knockdown of EIF4EBP1 suppressed the proliferation, migration and invasion abilities in ccRCC cells upon colony formation assays, wound healing assays and transwell assays (Fig. 6A-D).

Notably, expression of miR-125a-5p and *EIF4EBP1* were also verified in the TCGA dataset and the SYSU patient cohort (Fig. S1), and the interaction between them was also tested (Fig. S1). Consistent with previous reports [25], our data strongly suggest that *EIF4EBP1* acts as an oncogene in ccRCC cells, and that miR-125a-5p suppresses proliferation, migration, and invasion of ccRCC cells by targeting *EIF4EBP1.*

### circCHST15 attenuates the tumor-suppressive effect of miR-125a-5p in ccRCC cells

**Fig. 7:**
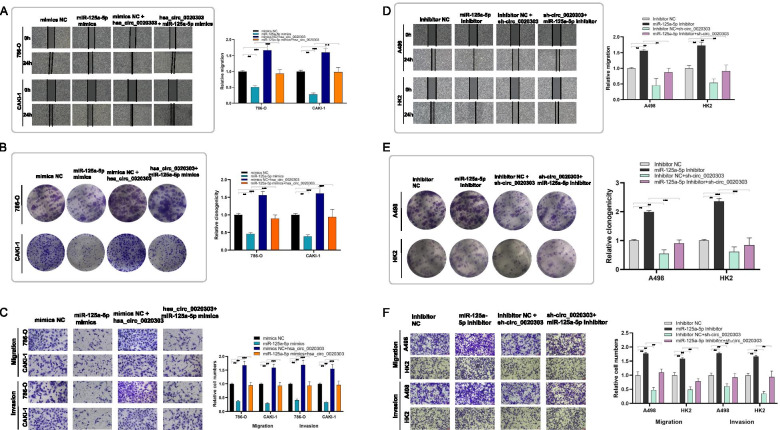
circCHST15 reverses the tumor-suppressing effect of miR-125a-5p on RCC cells in vitro. **A** Colony formation assay indicated that cell proliferation ability of 786-O and CAKI-1 cells transfected with miR-125a-5p mimics was reversed when co-transfected with circCHST15. **B** Wound healing assay indicated that cell migration capability of 786-O and CAKI-1 cells transfected with miR-125a-5p mimics was reversed when co-transfected with circCHST15. **C** Transwell migration and matrigel invasion assays demonstrated that cell migration and invasion abilities of 786-O and CAKI-1 cells transfected with miR-125a-5p mimics were counteracted when co-transfected with circCHST15. **D-F** The cell proliferation ability, cell migration capability, cell migration and invasion abilities of A498 and HK2 cells transfected with miR-125a-5p Inhibitor were counteracted when co-transfected with sh-circCHST15

**Fig. 8:**
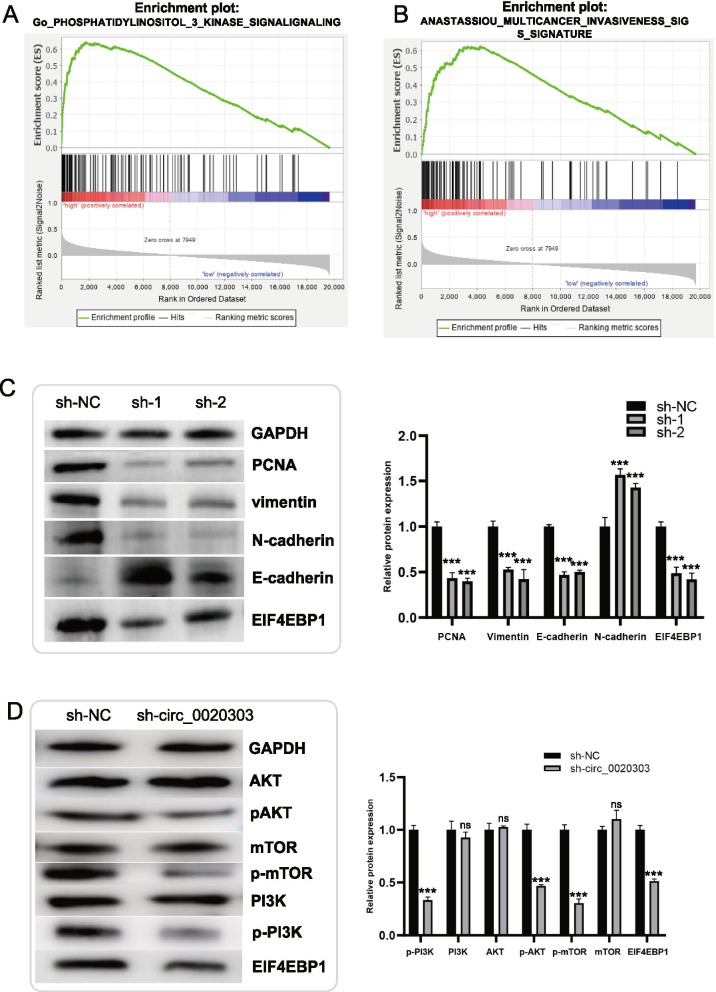
GSEA analysis and Western blot analysis. **A-B** GSEA analysis in TCGA patients with high and low EIF4EBP1 expression. **C-D** Western blot analysis indicated that circCHST15 promotes the ccRCC progression through PI3K-AKT-mTOR pathway. Histogram for protein expressions among different groups. *, *p* < 0.05; **, *p* < 0.01; ***, *p* < 0.001

**Fig. 9:**
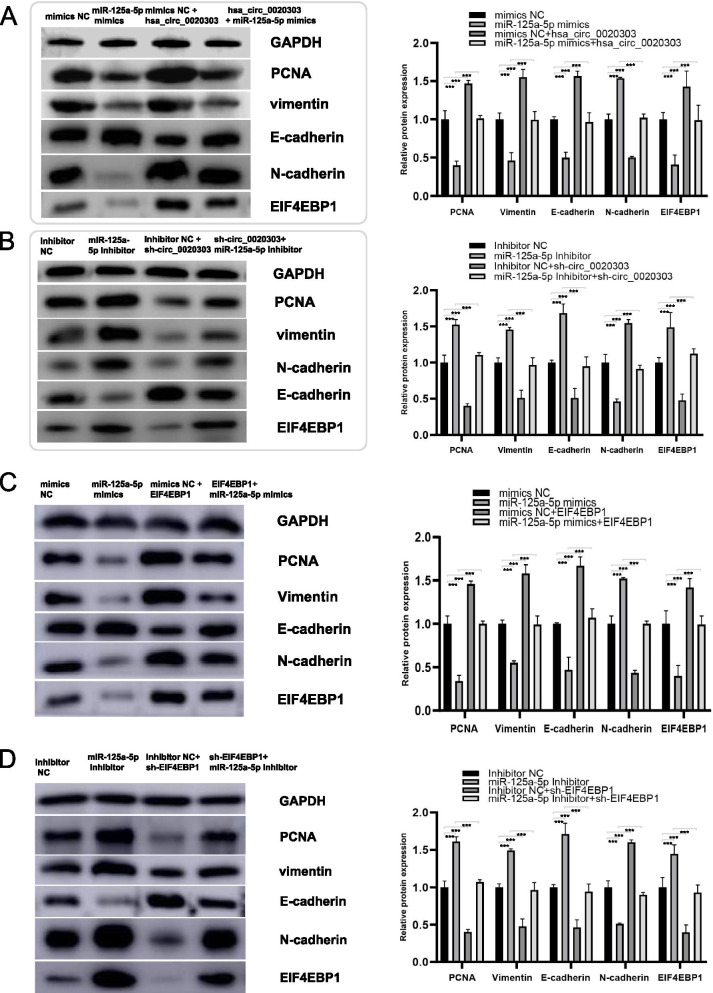
**A-B** Western blot analysis demonstrated that circCHST15/sh-circCHST15 could counteract the influence of miR-125a-5p mimics/Inhibitor on EIF4EBP1, PCNA, Vimentin, E-cadherin and N-cadherin expression in RCC cells. **C-D** Western blot analysis demonstrated that EIF4EBP1/sh-EIF4EBP1 could counteract the influence of miR-125a-5p mimics/Inhibitor on EIF4EBP1, PCNA, Vimentin, E-cadherin and N-cadherin expression in RCC cells. Histogram for protein expressions among different groups. *, *p* < 0.05; **, *p* < 0.01; ***, *p* < 0.001

Rescue experiments were performed in ccRCC cells co-transfecting circCHST15 and miR-125a-5p mimics, compared to cells transfected only with miR-125a-5p mimics. And it was observed that up-regulation of circCHST15, to some extent, could antagonize the weakened proliferation, migration and invasion abilities induced by miR-125a-5p in 786-O and CAKI-1 cells (Fig. 7A-C). On the other hand, downregulation of circCHST15 also lessened the miR-125a-5p inhibitor-mediated enhancement of migration and invasion in A498 and HK2 cells (Fig. 7D–F). According to the results of gene set enrichment analysis (GSEA), there are two main downstream pathways of *EIF4EBP1*: the *PI3K* pathway and the Anastassiou multicancer invasiveness pathway (Fig. 8A,B). Western blot analysis indicated substantially decreased expression of EIF4EBP1, PCNA, N-cadherin, and vimentin after knockdown of circCHST15, whereas E-cadherin was upregulated (Fig. 8C,D). In addition, western blot analysis showed increased EIF4EBP1 levels in ccRCC cells co-transfected with miR-125a-5p mimics and circCHST15 compared to ccRCC cells transfected only with miR-125a-5p mimics (Fig. 9A,B). Collectively, these results suggest that circCHST15 promotes ccRCC cell progression partly through mitigating the tumor suppression activity of miR-125a-5p.

### EIF4EBP1 weakens the tumor-suppressive effect of miR-125a-5p in ccRCC cells

To establish whether miR-125a-5p exerts its anti-cancer effect in ccRCC cells through targeting *EIF4EBP1*, rescue experiments were performed by co-transfecting *EIF4EBP1* and miR-125a-5p mimics in ccRCC cells. We observed enhanced colony formation ability in ccRCC cells co-transfected with miR-125a-5p mimics and *EIF4EBP1* compared to that of ccRCC cells transfected only with miR-125a-5p mimics, suggesting that overexpression of *EIF4EBP1* could partly rescue the reduced proliferative capacity phenotype induced by miR-125a-5p (Fig. S4B). Similarly, upregulation of *EIF4EBP1* partly attenuated the miR-125a-5p mimics-mediated suppression of migration and invasion in 786-O and CAKI-1 cells (Fig. S4A, C). On the other hand, EIF4EBP1 downregulation attenuated the miR-125a-5p inhibitor-mediated enhancement of migration and invasion in A498 and HK2 cells (Fig. S4D–F). Western blot analysis indicated that the expression of EIF4EBP1, PCNA, N-cadherin, and vimentin protein was significantly decreased after knockdown of *EIF4EBP1* or transfection with miR-125a-5p mimics, whereas E-cadherin was upregulated (Fig. 9C,D). Western blot analysis also indicated that EIF4EBP1, PCNA, N-cadherin, and vimentin levels were partly increased in ccRCC cells co-transfected with miR-125a-5p mimics and EIF4EBP1 compared to ccRCC cells transfected only with miR-125a-5p mimics (Fig. 9C,D). Collectively, these results suggest that miR-125a-5p suppresses ccRCC cell progression partly through targeting EIF4EBP1.

### cirCHST15 promotes tumor growth and metastasis in vivo

**Fig. 10:**
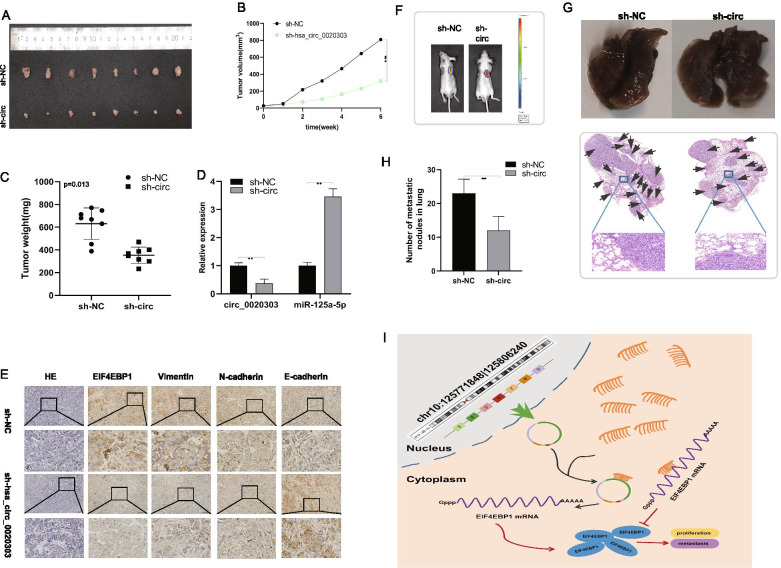
Knockdown of circCHST15 inhibits the growth and metastasis of RCC cells in vivo. **A-D** 786-O cells stably transfected with sh-control or sh-circCHST15 were injected subcutaneously into the left axilla of BALB/c nude mice. Tumor volume and weight were dramatically decreased in circCHST15 knockdown group. **E** HE staining and IHC analysis of EIF4EBP1, Vimentin, N-cadherin and E-cadherin expression in subcutaneous xenograft tumors. Knockdown of circCHST15 could up-regulate E-cadherin and down-regulate EIF4EBP1, Vimentin and N-cadherin expression. **F** Bioluminescence of the lung metastatic nodules was detected by an in vivo bioluminescence imaging system. **G** Representative images of HE staining analysis of the lung metastatic nodules in each group. **H** lung metastatic nodules were enucleated and measured. The numbers of lung metastatic nodules were significantly decreased in circCHST15 knockdown group. **I** Schematic diagram shows that circCHST15 promotes RCC cells proliferation and metastasis through miR-125a-5p/EIF4EBP1 axis

To investigate the effect of circCHST15 on tumor growth in vivo, 786-O cells stably transfected with sh-circCHST15 or control vector were subcutaneously injected into BALB/c nude mice. Tumor growth was monitored weekly for 4 weeks, at which time the study was terminated, and postmortem tumor assessments were performed. Tumor volume and weight were significantly decreased in the circCHST15 knockdown group compared to those of the vector group (Fig. 10A–D). Immunohistochemical analysis showed that expression of EIF4EBP1, vimentin, and N-cadherin was decreased in tumors derived from circCHST15-depleted cells compared to controls, whereas the E-cadherin expression level was markedly increased in circCHST15-depleted tumors (Fig. 10E).

To study the effects of circCHST15 knockdown in a model of ccRCC metastasis, we transfected firefly luciferase into our 786-O cells previously engineered for xenograft studies, and those cells were injected into the tail vein of nude mice. In circCHST15-silenced tumors, bioluminescence in the lung was weak or undetectable, and, upon postmortem examination, the number of nodules of lung metastases were decreased compared to animals with circCHST15-proficient tumors, suggesting that downregulation of circCHST15 suppresses lung metastasis of ccRCC in vivo (Fig. 10F–H). We also showed that miR-125a-5p mimics could rescue the effect of circCHST15 on renal tumor growth and metastasis (Figs. S5, S6).

## Discussion

With the increasing application of bioinformatics analysis and high-throughput sequencing, plenty of circRNAs were identified and proven to take an important role in the development and progression of various cancers [9, 10, 12, 14, 15, 26–29], including ccRCC [8, 10, 29]. However, the functions of circRNAs in ccRCC, and how they might be leveraged for clinical applications, remain largely unknown.

In this study, we identified a new circRNA, circCHST15, originating from exons 3, 4, 5, 6, 7, 8, and 9 of its host gene *CHST15*, which was upregulated in ccRCC cells and tissue. The existing literature suggests that CHST15 exerts oncogenic effects in pancreatic cancer stroma [30, 31], esophageal cancer [32], breast cancer [33], and ovarian cancer [34]. Additionally, these studies suggest that CHST15 can promote tumor progression by driving the proliferation and invasion of cancer cells, suggesting that high CHST15 might be associated with poor prognosis. According to our study, it was concluded that silencing of circCHST15 significantly suppressed the progression of ccRCC cells, whereas overexpression of circCHST15 had the opposite effect. And it was increasingly implied that circRNAs act as miRNA sponges in many cancer development18,25,34, thereby regulating downstream target genes. For instance, Circular RNA circ-ZKSCAN1 inhibits bladder cancer progression through miR-1178-3p/p21 axis and acts as a prognostic factor of recurrence [35]. According to our study, it was concluded that silencing of circCHST15 significantly suppressed the progression of ccRCC cells, and overexpressed circCHST15 acts inversely. And it was increasingly implied that circRNAs serve as miRNA sponges in cancer development [19, 26, 36], thereby regulating downstream target genes. For instance, circ-ZKSCAN1 inhibits bladder cancer progression through miR-1178-3p/p21 axis and acts as a prognostic factor of recurrence. Moreover, because circRNA is enriched in the cytoplasm, it was likely to play a role of ceRNA [9, 19, 37]. Our study confirmed that circCHST15 was mainly located in the cytoplasm through Cytoplasmic & Nuclear RNA Extraction assays and FISH assay. Besides, we performed RNA pull-down and dual-luciferase reporter assays, the results showed that there is an interaction between circCHST15 and miR-125a-5p in ccRCC cells. Subsequently, the functional effects of miR-125a-5p were assessed by regulating its’ expression in ccRCC cells. It was showed that miR-125a-5p exerted an anti-tumor role in ccRCC cells. Additionally, overexpression of circCHST15 alleviated miR-125a-5p-mediated suppression of cell proliferation, migration, and invasion in ccRCC cells. Taken together, circCHST15 performed its’ biological function by interacting with miR-125a-5p.

It was reported that miRNAs can bind to the 3′ UTR of target mRNAs to reduce expression level of this target gene [38]. And recently, it was indicated that circRNAs can regulate gene expression by directly binding to miRNAs to prevent them from interacting with target genes [8, 10, 28, 29, 37]. Our study identified that circCHST15 promoted ccRCC cells proliferation and metastasis through miR-125a-5p-mediated EIF4EBP1. Although EIF4EBP1 had been proven to act as an oncogene in some tumors [22–25], its biological effect in ccRCC has not been discovered. Thus, our study just delves into this in ccRCC cells.

Recent studies have indicated that circRNAs play a crucial role in the progression and prognosis of human cancer [36, 39], and the involvement of circRNAs in ccRCC has been investigated in several studies. For instance, circ-AKT3 inhibits ccRCC metastasis via altering miR-296-3p/E-cadherin signals, and patients with ccRCC with low circ- AKT3 had shortened survival [13]. circPTCH1 promotes RCC metastasis via the miR-485-5p/MMP14 axis and activation of the epithelial–mesenchymal transition process, and upregulated expression of circPTCH1 was positively correlated with advanced stage and worse survival in patients with ccRCC [11]. In our study, we showed that high expression of circCHST15 was associated with advanced pathological stage and poor survival, including OS and PFS.

## Conclusions

Our results showed that circCHST15 is an “oncogene” in ccRCC. Highly circCHST15 promote cell proliferation and metastasis through miR-125a-5p-mediated EIF4EBP1 (Fig. 10I). Our findings not only explain the mechanisms of circCHST15 in regulating ccRCC cells progression, but also provide a potential biomarker and therapeutic target for the management of ccRCC.

## Supplementary Information

**Additional file 1: Table S1.** The primers used in this study are listed as follows.

**Additional file 2: Table S2.** The oligonucleotides transfected in this study are listed as follows.

**Additional file 3: Table S3.** The probes used in this study are listed as follows.

**Additional file 4: Figure S1.** circCHST15 is associated with prognosis of RCC patients and exerts oncogenic effects through miR-125a-5p/EIF4EBP1 axis. **A**. Different expression of miR-125a-5p in ccRCC tissues compared with normal tissues in TCGA database. **B**. Different expression of EIF4EBP1 in ccRCC tissues compared with normal tissues in TCGA database. **C**. The expression of miR-125a-5p in 175 pairs of RCC tissues and adjacent normal tissues was detected by qRT-PCR analysis. **D**. The expression of EIF4EBP1 in 175 pairs of RCC tissues and adjacent normal tissues was detected by qRT-PCR analysis. **E**. Correlation analysis between circCHST15 and miR-125a-5p from SYSU cohort (*n* = 175). **F**. Correlation analysis between EIF4EBP1 and miR-125a-5p from SYSU cohort (*n* = 175). **A,C**:Kaplan-Meier survival curve demonstrated that high miR-125a-5p expression was correlated with low overall survival/progression-free survival of RCC patients in TCGA database and SYSU cohort. **B,D**: Kaplan-Meier survival curve demonstrated that low EIF4EBP1 expression was correlated with low overall survival/progression-free survival of RCC patients in TCGA database and SYSU cohort.

**Additional file 5: Figure S2.** Comparison of the circCHST15 with other known clinical prognostic biomarkers:ROC analyses of different prognostic biomarkers. A,C based on patients’ OS B, D based on patients’ PFS. A-B, To compare the circCHST15 with other known prognostic biomarkers. C-D, To compare the circCHST15 with clinical prognostic score algorithm(SSIGN score).

**Additional file 6: Figure S3.** Norther blot, qRT-PCR assay and RNA pull-down assay were performed in ccRCC cells. **A-B**, Norther blot of circCHST15 was provided to validate the existence of the endogenous circRNA. **C**, We took qRT-PCR assay in HK2, 786-O, CAKI-1 and A498 to test the relative expression of four miRNAs. **D**, we still used the wildtype 786-O and CAKI-1 cells to perform that the biotin-labeled miR-125a-5p still capture the circCHST15.

**Additional file 7: Figure S4.** EIF4EBP1 reverses the tumor-suppressing effect of miR-125a-5p on RCC cells in vitro. **A**. Colony formation assay indicated that cell proliferation ability of 786-O and CAKI-1 cells transfected with miR-125a-5p mimics was reversed when co-transfected with EIF4EBP1. **B**. Wound healing assay indicated that cell migration capability of 786-O and CAKI-1 cells transfected with miR-125a-5p mimics was reversed when co-transfected with EIF4EBP1. **C**. Transwell migration and matrigel invasion assays demonstrated that cell migration and invasion abilities of 786-O and CAKI-1 cells transfected with miR-125a-5p mimics were counteracted when co-transfected with EIF4EBP1. **D-F**. The cell proliferation ability, cell migration capability, cell migration and invasion abilities of A498 and HK2 cells transfected with miR-125a-5p Inhibitor were counteracted when co-transfected with sh-EIF4EBP1.

**Additional file 8: Figure S5.** circCHST15 reverses the tumor-suppressing effect of miR-125a-5p on the growth of RCC cells(786-O) in vivo. **A-D**. These BALB/c nude mice were divided into four groups and were treated separately. Tumor volume and weight were dramatically decreased in agomir-miR-125a-5p group. However, circCHST15 reverses the tumor-suppressing effect of miR-125a-5p on the growth of 786-O cells in vivo.

**Additional file 9: Figure S6.** circCHST15 reverses the tumor-suppressing effect of miR-125a-5p on the metastasis of RCC cells in vivo. **A**. Bioluminescence of the lung metastatic nodules was detected by an in vivo bioluminescence imaging system. **B**. Representative images of HE staining analysis of the lung metastatic nodules in each group. **C**. lung metastatic nodules were enucleated and measured. The numbers of lung metastatic nodules were significantly decreased in agomir-miR-125a-5p group. However, circCHST15 reverses the tumor-suppressing effect of miR-125a-5p on the metastasis of 786-O cells in vivo. **D**. HE staining and IHC analysis of EIF4EBP1, Vimentin, N-cadherin and E-cadherin expression in subcutaneous xenograft tumors. circCHST15 reverses the tumor-suppressing effect of miR-125a-5p on the proteins’ expression of E-cadherin, EIF4EBP1, Vimentin and N-cadherin.

## Data Availability

The RNA-seq data of human RCC tissues and normal kidney tissues during this study are included in the uploaded GEO datasets (GSE100186 and GSE137836). The rest of datasets used and analyzed during the current study are available from the corresponding author on reasonable request.

## Abbreviations

ccRCC: Clear cell renal cell carcinoma
UTR: Untranslated region
CHST15: Carbohydrate sulfotransferase 15
circRNA: Circular RNA
EIF4EBP1: Eukaryotic translation initiation factor 4E binding protein 1
qRT–PCR: Quantitative real-time polymerase chain reaction
miRNA: Micro RNA
FISH: Fluorescence in situ hybridization
GEO: Gene Expression Omnibus
OS: Overall survival
PFS: Progression-free survival
TCGA: The Cancer Genome Atlas
SYSU: Sun Yat-Sen University

## References

[1] Scelo G, Larose TL. Epidemiology and Risk Factors for Kidney Cancer. J Clin Oncol. 2018;36(36):JCO2018791905. 10.1200/JCO.2018.79.1905. 10.1200/JCO.2018.79.1905 PMC6299342 30372394

[2] Siegel RL, Miller KD, Fuchs HE, Jemal A (2021). Cancer statistics, 2021. CA Cancer J Clin.

[3] Powles T, Staehler M, Ljungberg B, Bensalah K, Canfield SE, Dabestani S, Giles RH, Hofmann F, Hora M, Kuczyk MA, Lam T, Marconi L, Merseburger AS, Volpe A, Bex A (2016). European Association of Urology guidelines for clear cell renal cancers that are resistant to vascular endothelial growth factor receptor-targeted therapy. Eur Urol.

[4] Powles T, Staehler M, Ljungberg B, Bensalah K, Canfield SE, Dabestani S, Giles R, Hofmann F, Hora M, Kuczyk MA, Lam T, Marconi L, Merseburger AS, Volpe A, Bex A (2016). Updated EAU guidelines for clear cell renal Cancer patients who fail VEGF targeted therapy. Eur Urol.

[5] Memczak S, Jens M, Elefsinioti A, Torti F, Krueger J, Rybak A, Maier L, Mackowiak SD, Gregersen LH, Munschauer M, Loewer A, Ziebold U, Landthaler M, Kocks C, le Noble F, Rajewsky N (2013). Circular RNAs are a large class of animal RNAs with regulatory potency. Nature.

[6] Salzman J, Chen RE, Olsen MN, Wang PL, Brown PO (2013). Cell-type specific features of circular RNA expression. PLoS Genet.

[7] Jeck WR, Sorrentino JA, Wang K, Slevin MK, Burd CE, Liu J, Marzluff WF, Sharpless NE (2013). Circular RNAs are abundant, conserved, and associated with ALU repeats. RNA.

[8] Chen Z, Xiao K, Chen S, Huang Z, Ye Y, Chen T (2020). Circular RNA hsa_circ_001895 serves as a sponge of microRNA-296-5p to promote clear cell renal cell carcinoma progression by regulating SOX12. Cancer Sci.

[9] Hong W, Xue M, Jiang J, Zhang Y, Gao X (2020). Circular RNA circ-CPA4/ let-7 miRNA/PD-L1 axis regulates cell growth, stemness, drug resistance and immune evasion in non-small cell lung cancer (NSCLC). J Exp Clin Cancer Res.

[10] Li J, Huang C, Zou Y, Ye J, Yu J, Gui Y (2020). CircTLK1 promotes the proliferation and metastasis of renal cell carcinoma by sponging miR-136-5p. Mol Cancer.

[11] Liu H, Hu G, Wang Z, Liu Q, Zhang J, Chen Y, Huang Y, Xue W, Xu Y, Zhai W (2020). circPTCH1 promotes invasion and metastasis in renal cell carcinoma via regulating miR-485-5p/MMP14 axis. Theranostics.

[12] Liu Z, Zhou Y, Liang G, Ling Y, Tan W, Tan L, Andrews R, Zhong W, Zhang X, Song E, Gong C (2019). Circular RNA hsa_circ_001783 regulates breast cancer progression via sponging miR-200c-3p. Cell Death Dis.

[13] Xue D, Wang H, Chen Y, Shen D, Lu J, Wang M, Zebibula A, Xu L, Wu H, Li G, Xia L (2019). Circ-AKT3 inhibits clear cell renal cell carcinoma metastasis via altering miR-296-3p/E-cadherin signals. Mol Cancer.

[14] Wang Y, Yin L, Sun X (2020). CircRNA hsa_circ_0002577 accelerates endometrial cancer progression through activating IGF1R/PI3K/Akt pathway. J Exp Clin Cancer Res.

[15] Yao Y, Chen X, Yang H, Chen W, Qian Y, Yan Z, Liao T, Yao W, Wu W, Yu T, Chen Y, Zhang Y (2019). Hsa_circ_0058124 promotes papillary thyroid cancer tumorigenesis and invasiveness through the NOTCH3/GATAD2A axis. J Exp Clin Cancer Res.

[16] Zhang Y, Liang W, Zhang P, Chen J, Qian H, Zhang X, Xu W (2017). Circular RNAs: emerging cancer biomarkers and targets. J Exp Clin Cancer Res.

[17] Irizarry RA, Hobbs B, Collin F, Beazer-Barclay YD, Antonellis KJ, Scherf U, Speed TP (2003). Exploration, normalization, and summaries of high density oligonucleotide array probe level data. Biostatistics.

[18] Chen LL (2016). The biogenesis and emerging roles of circular RNAs. Nat Rev Mol Cell Biol.

[19] Hansen TB, Jensen TI, Clausen BH, Bramsen JB, Finsen B, Damgaard CK, Kjems J (2013). Natural RNA circles function as efficient microRNA sponges. Nature.

[20] Wong N, Wang X (2015). miRDB: an online resource for microRNA target prediction and functional annotations. Nucleic Acids Res.

[21] Agarwal V, Bell GW, Nam JW, Bartel DP. Predicting effective microRNA target sites in mammalian mRNAs. Elife. 2015;4:e05005. 10.7554/eLife.05005. 10.7554/eLife.05005 PMC4532895 26267216

[22] Li SQ, Feng J, Yang M, Ai XP, He M, Liu F (2020). Sauchinone: a prospective therapeutic agent-mediated EIF4EBP1 down-regulation suppresses proliferation, invasion and migration of lung adenocarcinoma cells. J Nat Med.

[23] Cha YL, Li PD, Yuan LJ, Zhang MY, Zhang YJ, Rao HL, Zhang HZ, Zheng XF, Wang HY (2015). EIF4EBP1 overexpression is associated with poor survival and disease progression in patients with hepatocellular carcinoma. PLoS One.

[24] Gao W, Lam JW, Li JZ, Chen SQ, Tsang RK, Chan JY, Wong TS (2017). MicroRNA-138-5p controls sensitivity of nasopharyngeal carcinoma to radiation by targeting EIF4EBP1. Oncol Rep.

[25] Lee M, Kim EJ, Jeon MJ (2016). MicroRNAs 125a and 125b inhibit ovarian cancer cells through post-transcriptional inactivation of EIF4EBP1. Oncotarget.

[26] Kulcheski FR, Christoff AP, Margis R (2016). Circular RNAs are miRNA sponges and can be used as a new class of biomarker. J Biotechnol.

[27] Zhang M, Zhao K, Xu X, Yang Y, Yan S, Wei P, Liu H, Xu J, Xiao F, Zhou H, Yang X, Huang N, Liu J, He K, Xie K, Zhang G, Huang S, Zhang N (2018). A peptide encoded by circular form of LINC-PINT suppresses oncogenic transcriptional elongation in glioblastoma. Nat Commun.

[28] Liu H, Bi J, Dong W, Yang M, Shi J, Jiang N, Lin T, Huang J (2018). Invasion-related circular RNA circFNDC3B inhibits bladder cancer progression through the miR-1178-3p/G3BP2/SRC/FAK axis. Mol Cancer.

[29] Chen Q, Liu T, Bao Y, Zhao T, Wang J, Wang H, Wang A, Gan X, Wu Z, Wang L (2020). CircRNA cRAPGEF5 inhibits the growth and metastasis of renal cell carcinoma via the miR-27a-3p/TXNIP pathway. Cancer Lett.

[30] Nishimura M, Matsukawa M, Fujii Y, Matsuda Y, Arai T, Ochiai Y, Itoi T, Yahagi N (2018). Effects of EUS-guided intratumoral injection of oligonucleotide STNM01 on tumor growth, histology, and overall survival in patients with unresectable pancreatic cancer. Gastrointest Endosc.

[31] Matsuda Y, Fujii Y, Matsukawa M, Ishiwata T, Nishimura M, Arai T (2019). Overexpression of carbohydrate sulfotransferase 15 in pancreatic cancer stroma is associated with worse prognosis. Oncol Lett.

[32] Wang X, Cheng G, Zhang T, Deng L, Xu K, Xu X, Wang W, Zhou Z, Feng Q, Chen D, Bi N, Wang L (2020). CHST15 promotes the proliferation of TE1 cells via multiple pathways in esophageal cancer. Oncol Rep.

[33] Liu LC, Wang YL, Lin PL, Zhang X, Cheng WC, Liu SH, Chen CJ, Hung Y, Jan CI, Chang LC, Qi X, Hsieh-Wilson LC, Wang SC (2019). Long noncoding RNA HOTAIR promotes invasion of breast cancer cells through chondroitin sulfotransferase CHST15. Int J Cancer.

[34] van der Steen SC, van Tilborg AA, Vallen MJ, Bulten J, van Kuppevelt TH, Massuger LF (2016). Prognostic significance of highly sulfated chondroitin sulfates in ovarian cancer defined by the single chain antibody GD3A11. Gynecol Oncol.

[35] Bi J, Liu H, Dong W, Xie W, He Q, Cai Z, Huang J, Lin T (2019). Circular RNA circ-ZKSCAN1 inhibits bladder cancer progression through miR-1178-3p/p21 axis and acts as a prognostic factor of recurrence. Mol Cancer.

[36] Zhong Y, Du Y, Yang X, Mo Y, Fan C, Xiong F, Ren D, Ye X, Li C, Wang Y, Wei F, Guo C, Wu X, Li X, Li Y, Li G, Zeng Z, Xiong W (2018). Circular RNAs function as ceRNAs to regulate and control human cancer progression. Mol Cancer.

[37] Zeng K, Chen X, Xu M, Liu X, Hu X, Xu T, Sun H, Pan Y, He B, Wang S (2018). CircHIPK3 promotes colorectal cancer growth and metastasis by sponging miR-7. Cell Death Dis.

[38] Bartel DP (2018). Metazoan MicroRNAs. Cell.

[39] Yang Z, Xie L, Han L, Qu X, Yang Y, Zhang Y, He Z, Wang Y, Li J (2017). Circular RNAs: regulators of Cancer-related signaling pathways and potential diagnostic biomarkers for human cancers. Theranostics.

