# Supplementary material for: circCHST15 is a novel prognostic biomarker that promotes clear cell renal cell carcinoma cell proliferation and metastasis through the miR-125a-5p/EIF4EBP1 axis
Source: Mol Cancer. 2021 Dec 18;20:169. doi: 10.1186/s12943-021-01449-w (PMC8684108; doi:10.1186/s12943-021-01449-w)
Supplement: Supplementary file 1 — Additional file 1: Table S1. The primers used in this study are listed as follows. [file 12943_2021_1449_MOESM1_ESM.docx]

|  | **Sequence (5’-3’)** |
| --- | --- |
| **Primers** | |
| Hsa_circ_0020303 Forward | GCTCTATGCTGTGTACCTTCTGGA |
| Hsa_circ_0020303 Reverse | TCTCATTGATGGATGGATTGCC |
| CHST15 Forward | TCGTGTGGACAGTAAGCAGAT |
| CHST15 Reverse | TGTAAGAAGCCATTACCAAGGTC |
| GAPDH Forward | GGAGCGAGATCCCTCCAAAAT |
| GAPDH Reverse | GGCTGTTGTCATACTTCTCATGG |
| EIF4EBP1 Forward | CTATGACCGGAAATTCCTGATGG |
| EIF4EBP1 Reverse | CCCGCTTATCTTCTGGGCTA |
| miR-125a-5p Forward | GGCGTCCCTGAGACCCTTTAA |
| miR-125b-5p Forward | CCGTCCCTGAGACCCTAACTT |
| miR-206 Forward | CCGAGGCCACATGCTTCTTTAT |
| miR-20b-5p Forward | CGCCAAAGUGCUCAUAGUG |
| miRNA Reverse | GTGCAGGGTCCGAGGT |
| U6 Forward | CGCTTCGGCAGCACATATAC |
| U6 Reverse | TTCACGAATTTGCGTGTCAT |
| miR-155-5p Forward | TTAATGCTAATCGTGATAGGG |
| miR-194-5p Forward | TGTAACAGCAACTCCATGTG |

**Table S1.** **The primers used in this study are listed as follows.**
