# Supplementary material for: circCHST15 is a novel prognostic biomarker that promotes clear cell renal cell carcinoma cell proliferation and metastasis through the miR-125a-5p/EIF4EBP1 axis
Source: Mol Cancer. 2021 Dec 18;20:169. doi: 10.1186/s12943-021-01449-w (PMC8684108; doi:10.1186/s12943-021-01449-w)
Supplement: Supplementary file 2 — Additional file 2: Table S2. The oligonucleotides transfected in this study are listed as follows. [file 12943_2021_1449_MOESM2_ESM.docx]

|  | **Sequence (5’-3’)** |
| --- | --- |
| **Oligonucleotides** | |
| si-NC sense | UUCUCCGAACGUGUCACGUTT |
| si-NC antisense | ACGUGACACGUUCGGAGAATT |
| circCHST15-si1 sense | AAAUUAAAAGAAAUCAUGGUC |
| circCHST15-si1 antisense | CCAUGAUUUCUUUUAAUUUUC |
| circCHST15-si2 sense | AAAAUUAAAAGAAAUCAUGGU |
| circCHST15-si2 antisense | CAUGAUUUCUUUUAAUUUUCU |
| EIF4EBP1-si1 sense | AGAAAGAGGAAACAAACGGGG |
| EIF4EBP1-si1 antisense | CCGUUUGUUUCCUCUUUCUGU |
| EIF4EBP1-si2 sense | ACAGAAAGAGGAAACAAACGG |
| EIF4EBP1-si2 antisense | GUUUGUUUCCUCUUUCUGUUA |
| mimics NC | AUUGGAACGAUACAG AGAAGAUU |
| miR-125a-5p mimics | UCCCUGAGACCCUUUAACCUGUGA |
| inhibitor NC | CAGUACUUUUGUGUAGUACAA |
| miR-125a-5p inhibitor | UCACAGGUUAAA GGGUCUCAGGGA |

**Table S2.** **The oligonucleotides transfected in this study are listed as follows.**
