# Supplementary material for: circCHST15 is a novel prognostic biomarker that promotes clear cell renal cell carcinoma cell proliferation and metastasis through the miR-125a-5p/EIF4EBP1 axis
Source: Mol Cancer. 2021 Dec 18;20:169. doi: 10.1186/s12943-021-01449-w (PMC8684108; doi:10.1186/s12943-021-01449-w)
Supplement: Supplementary file 3 — Additional file 3: Table S3. The probes used in this study are listed as follows. [file 12943_2021_1449_MOESM3_ESM.docx]

**Table S3. The probes used in this study are listed as follows.**

|  | **Sequence (5’-3’)** |
| --- | --- |
| **FISH Probes** | |
| Cy3-U6 | TTTGCGTGTCATCCTTGCG |
| Cy3-18S | CTTCCTTGGATGTGGTAGCCGTTTC |
| Cy3-circCHST15 | TTCCAGTTTCTGAACCTAGGAAATCTGGCATTTTTTAAAG |
| Cy5-miR-125a-5p | AGGTTAAAGGGTCTCAGGGA |
| **Biotin-coupled probes** | |
| Biotin-NC | CUGUACUUGUUCCAACUCAAGUGCUAUACUUGGUAGAUCAGA |
| Biotin-circCHST15 | UUCCAGUUUCUGAACCUAGGAAAUCUGGCAUUUUUUAAAG |
| Biotin-miR-125a-5p-wt | UCCCUGAGACCCUUUAACCU |
| Biotin-miR-125a-5p-mut | ACGACAGAGAAAAUGUCUU  UGGGACUCACCCUUUAACCU |
