# Supplementary figures and images for: circCHST15 is a novel prognostic biomarker that promotes clear cell renal cell carcinoma cell proliferation and metastasis through the miR-125a-5p/EIF4EBP1 axis
Source: Mol Cancer. 2021 Dec 18;20:169. doi: 10.1186/s12943-021-01449-w (PMC8684108; doi:10.1186/s12943-021-01449-w)

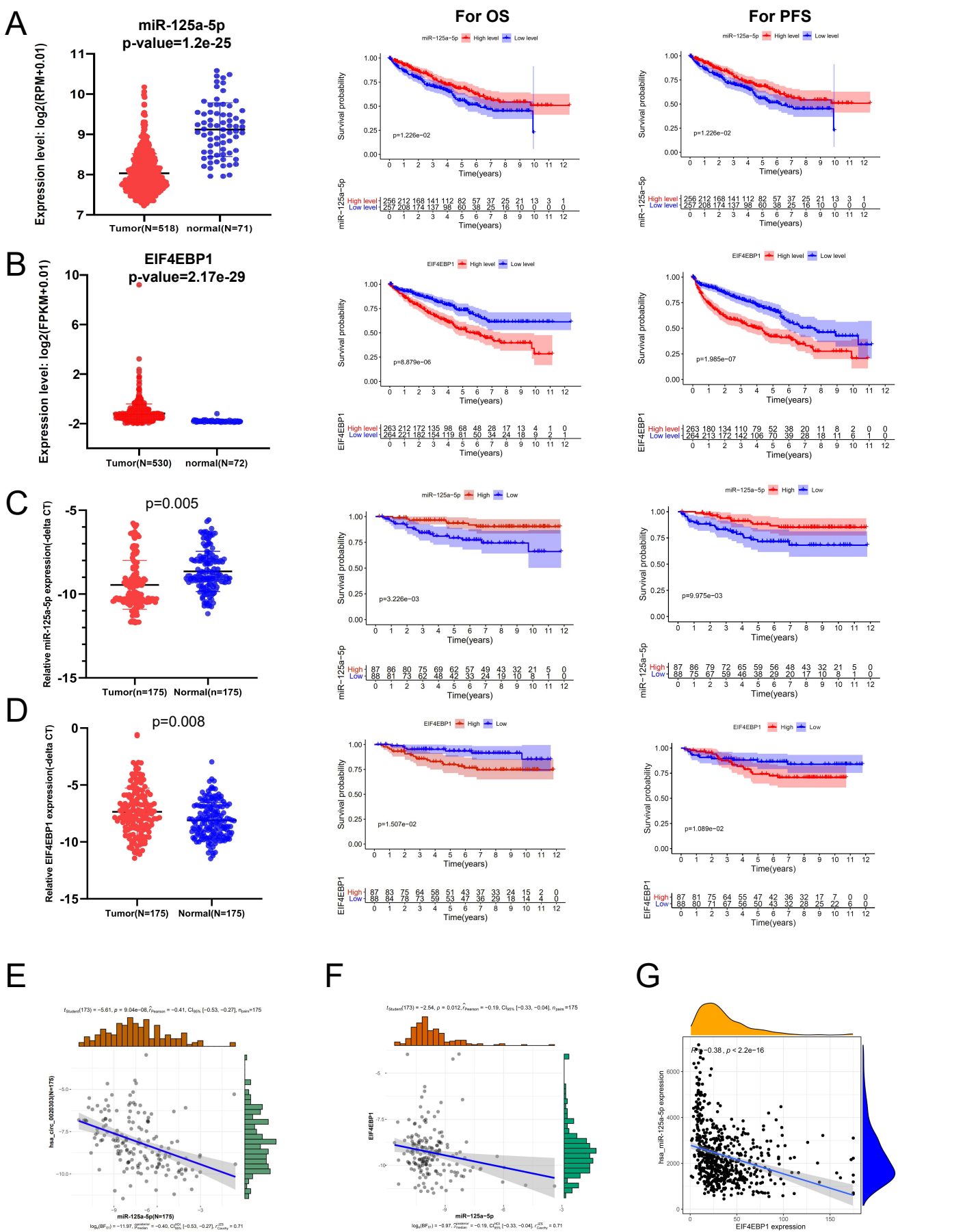

Supplement: Supplementary file 4 — Additional file 4: Figure S1. circCHST15 is associated with prognosis of RCC patients and exerts oncogenic effects through miR-125a-5p/EIF4EBP1 axis. A. Different expression of miR-125a-5p in ccRCC tissues compared with normal tissues in TCGA database. B. Different expression of EIF4EBP1 in ccRCC tissues compared with normal tissues in TCGA database. C. The expression of miR-125a-5p in 175 pairs of RCC tissues and adjacent normal tissues was detected by qRT-PCR analysis. D. The expression of EIF4EBP1 in 175 pairs of RCC tissues and adjacent normal tissues was detected by qRT-PCR analysis. E. Correlation analysis between circCHST15 and miR-125a-5p from SYSU cohort (n = 175). F. Correlation analysis between EIF4EBP1 and miR-125a-5p from SYSU cohort (n = 175). A,C:Kaplan-Meier survival curve demonstrated that high miR-125a-5p expression was correlated with low overall survival/progression-free survival of RCC patients in TCGA database and SYSU cohort. B,D: Kaplan-Meier survival curve demonstrated that low EIF4EBP1 expression was correlated with low overall survival/progression-free survival of RCC patients in TCGA database and SYSU cohort. [file 12943_2021_1449_MOESM4_ESM.pdf]

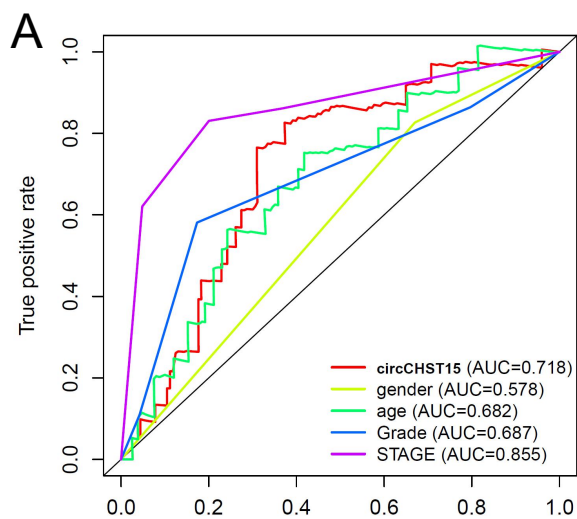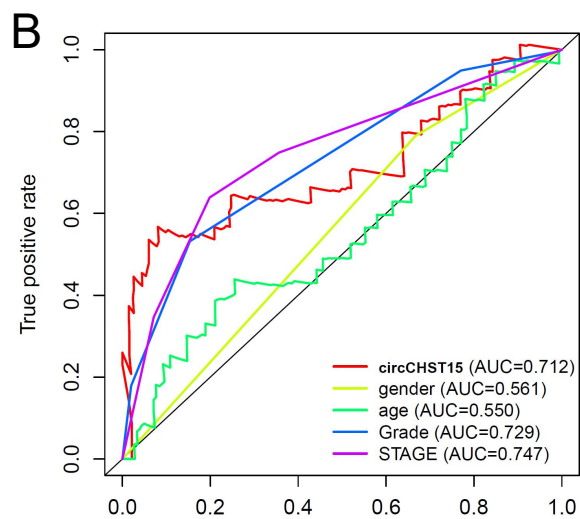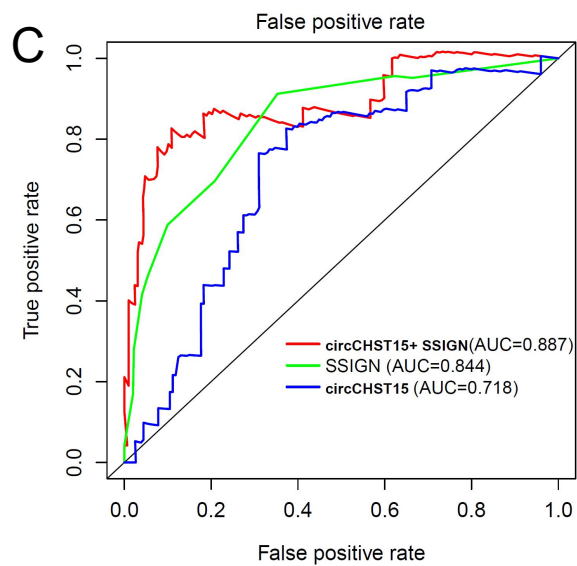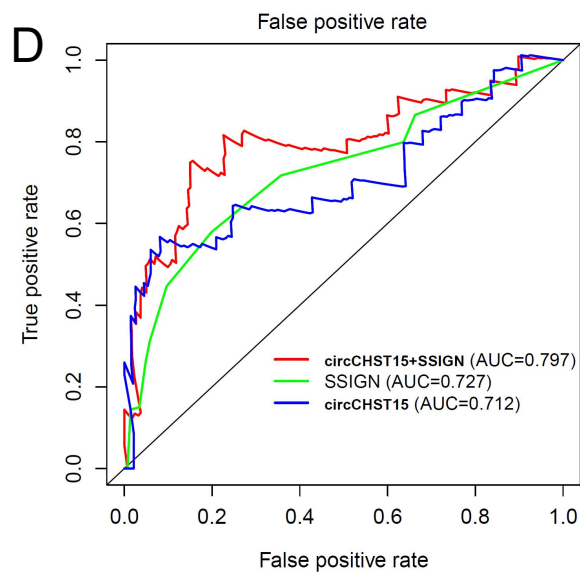

Supplement: Supplementary file 5 — Additional file 5: Figure S2. Comparison of the circCHST15 with other known clinical prognostic biomarkers:ROC analyses of different prognostic biomarkers. A,C based on patients’ OS B, D based on patients’ PFS. A-B, To compare the circCHST15 with other known prognostic biomarkers. C-D, To compare the circCHST15 with clinical prognostic score algorithm(SSIGN score). [file 12943_2021_1449_MOESM5_ESM.pdf]

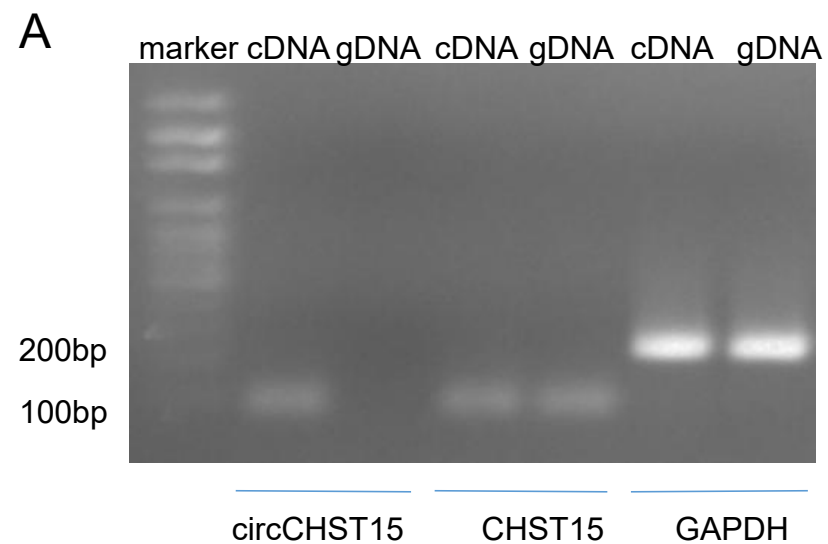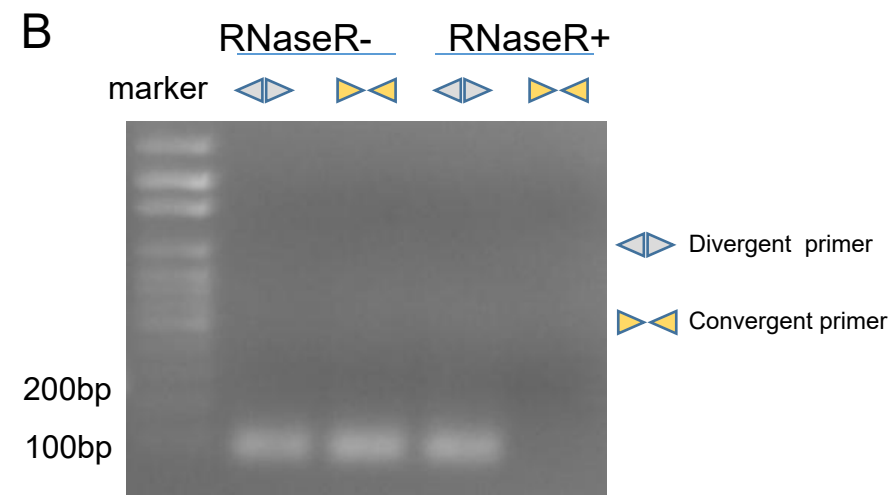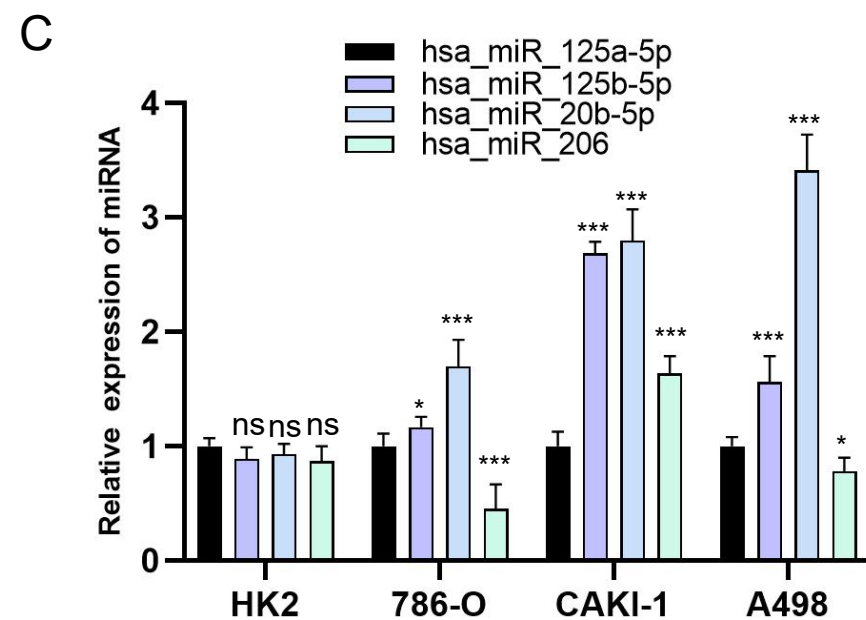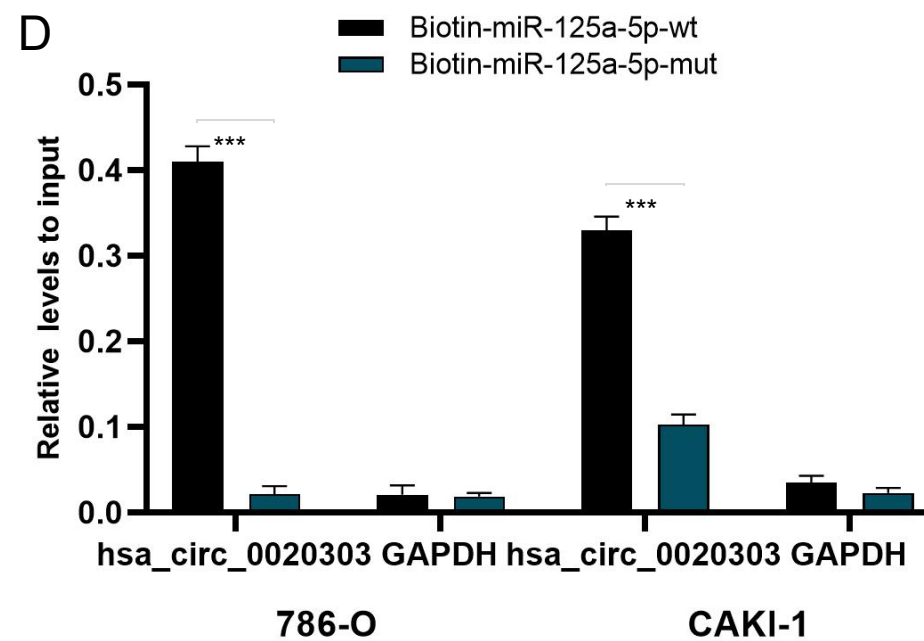

Supplement: Supplementary file 6 — Additional file 6: Figure S3. Norther blot, qRT-PCR assay and RNA pull-down assay were performed in ccRCC cells. A-B, Norther blot of circCHST15 was provided to validate the existence of the endogenous circRNA. C, We took qRT-PCR assay in HK2, 786-O, CAKI-1 and A498 to test the relative expression of four miRNAs. D, we still used the wildtype 786-O and CAKI-1 cells to perform that the biotin-labeled miR-125a-5p still capture the circCHST15. [file 12943_2021_1449_MOESM6_ESM.pdf]

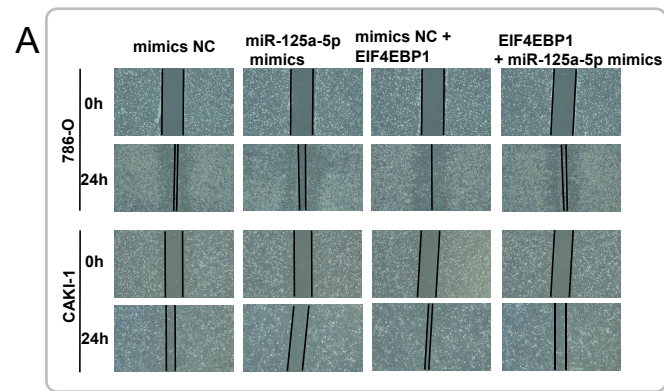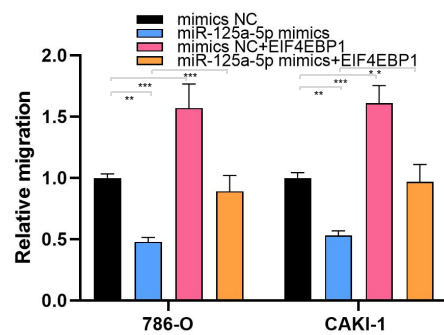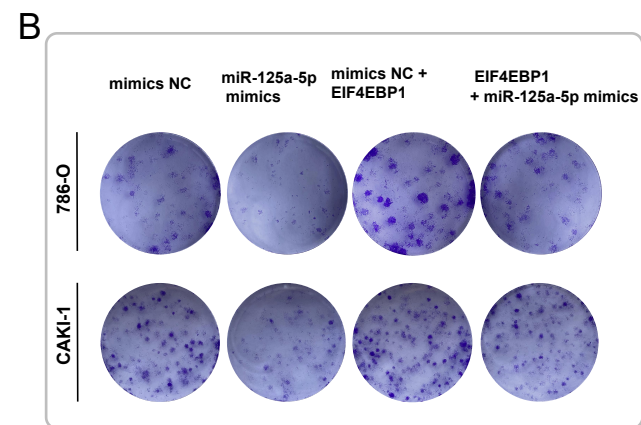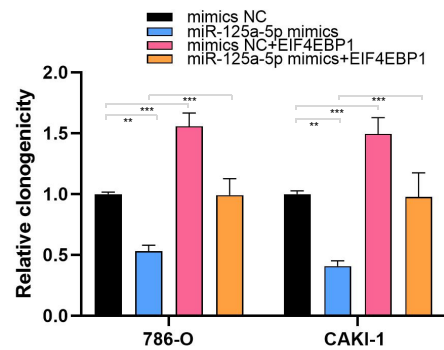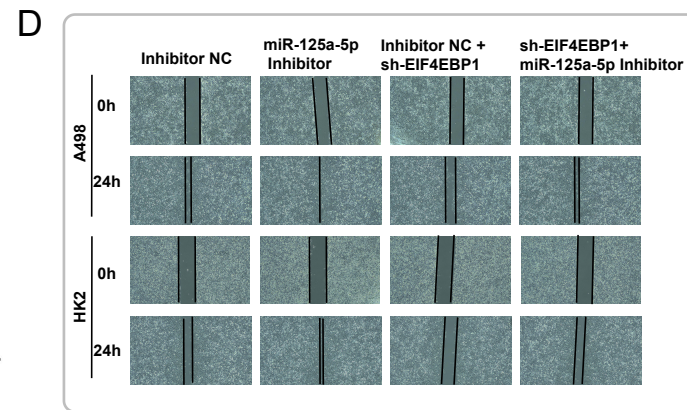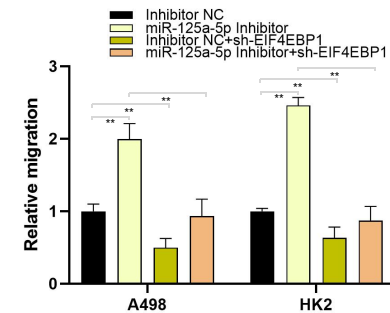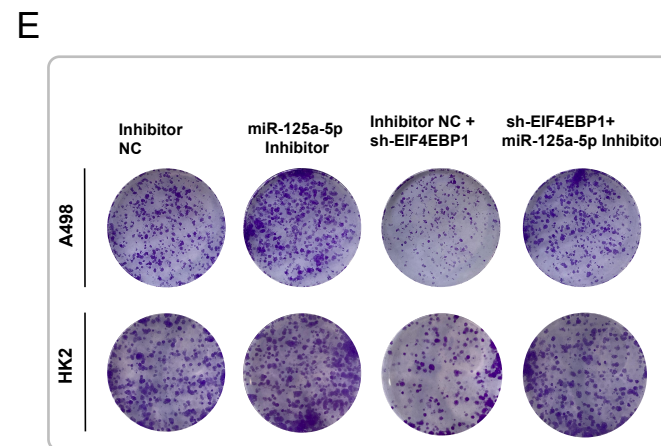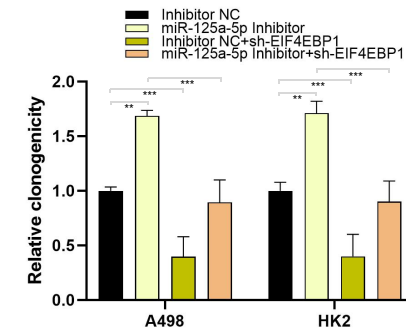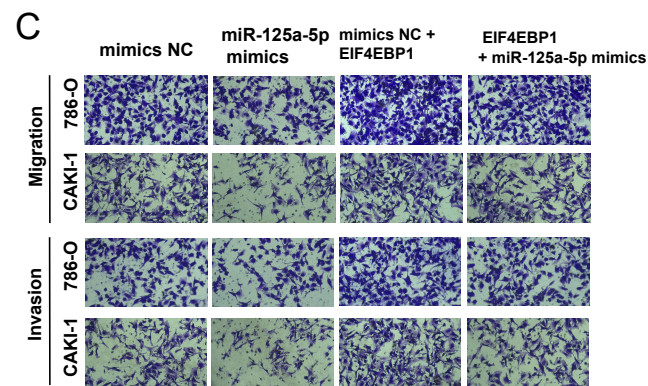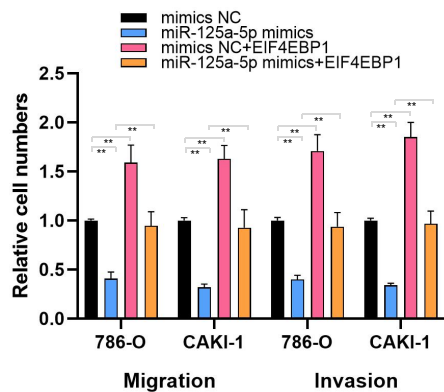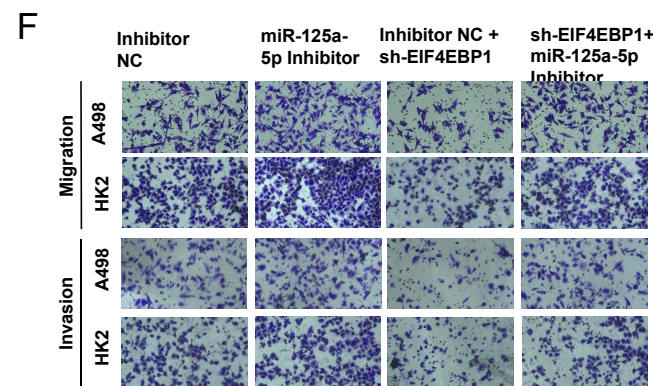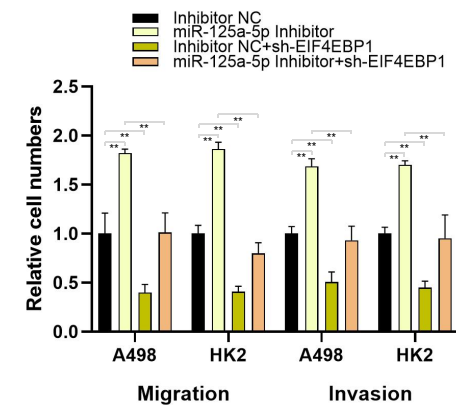

Supplement: Supplementary file 7 — Additional file 7: Figure S4. EIF4EBP1 reverses the tumor-suppressing effect of miR-125a-5p on RCC cells in vitro. A. Colony formation assay indicated that cell proliferation ability of 786-O and CAKI-1 cells transfected with miR-125a-5p mimics was reversed when co-transfected with EIF4EBP1. B. Wound healing assay indicated that cell migration capability of 786-O and CAKI-1 cells transfected with miR-125a-5p mimics was reversed when co-transfected with EIF4EBP1. C. Transwell migration and matrigel invasion assays demonstrated that cell migration and invasion abilities of 786-O and CAKI-1 cells transfected with miR-125a-5p mimics were counteracted when co-transfected with EIF4EBP1. D-F. The cell proliferation ability, cell migration capability, cell migration and invasion abilities of A498 and HK2 cells transfected with miR-125a-5p Inhibitor were counteracted when co-transfected with sh-EIF4EBP1. [file 12943_2021_1449_MOESM7_ESM.pdf]

A

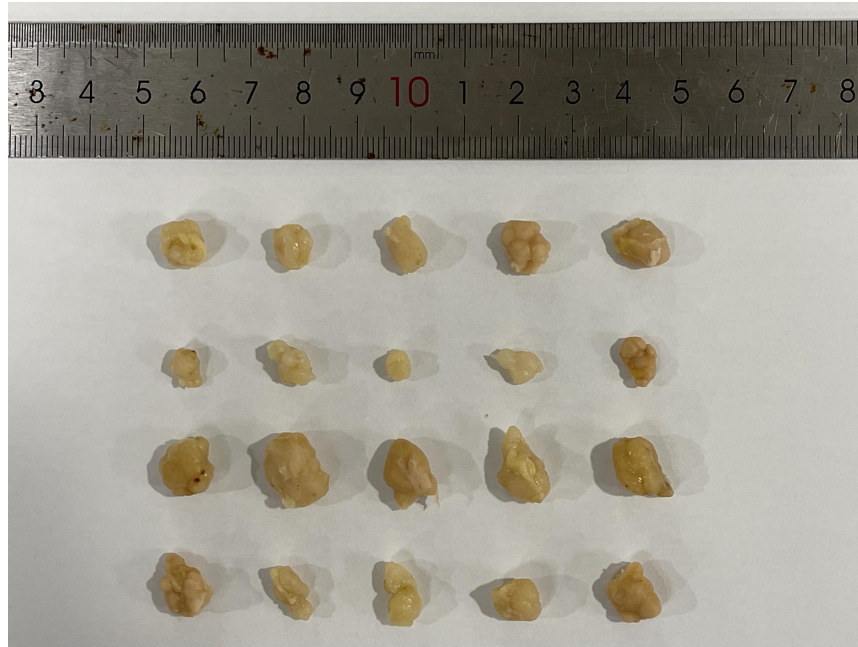

B

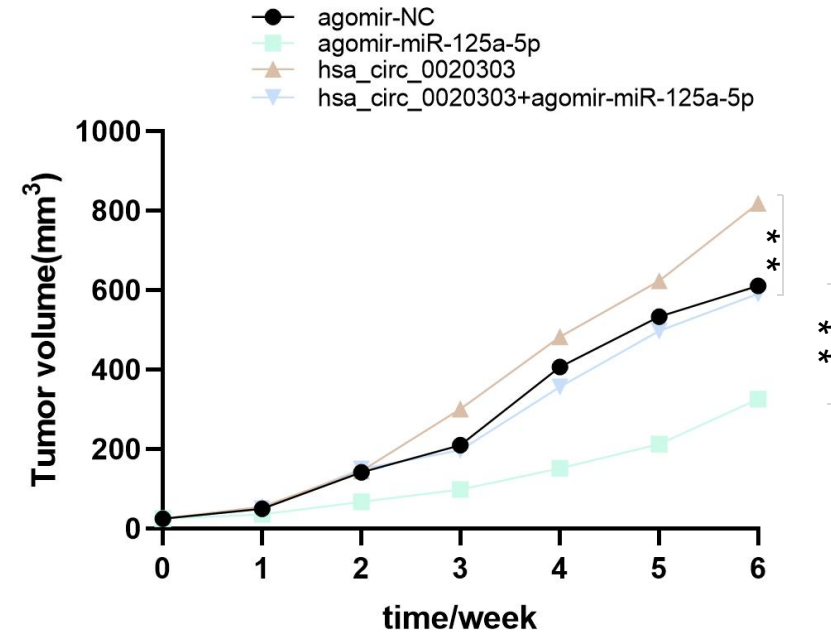

C

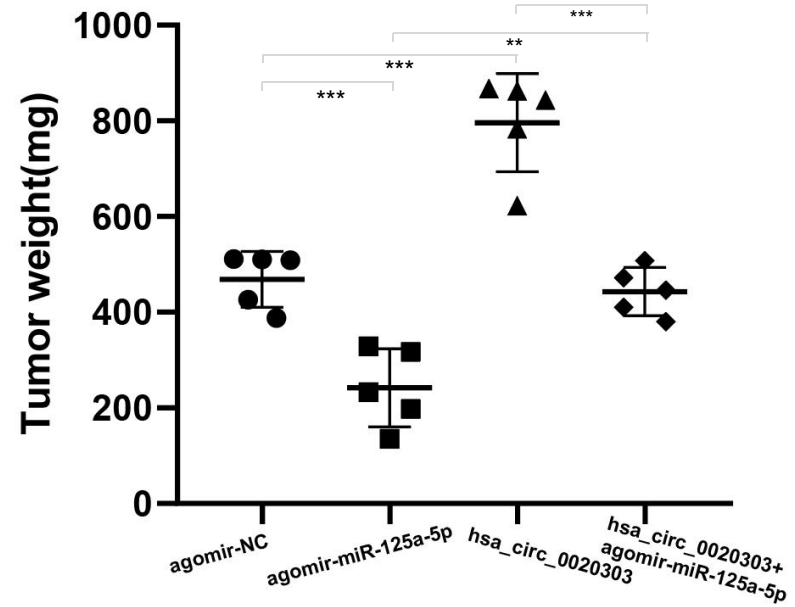

D

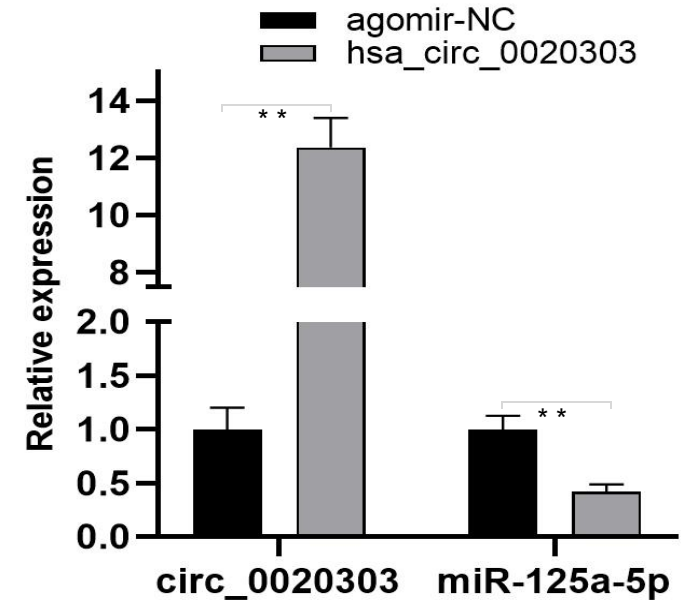

Supplement: Supplementary file 8 — Additional file 8: Figure S5. circCHST15 reverses the tumor-suppressing effect of miR-125a-5p on the growth of RCC cells(786-O) in vivo. A-D. These BALB/c nude mice were divided into four groups and were treated separately. Tumor volume and weight were dramatically decreased in agomir-miR-125a-5p group. However, circCHST15 reverses the tumor-suppressing effect of miR-125a-5p on the growth of 786-O cells in vivo. [file 12943_2021_1449_MOESM8_ESM.pdf]

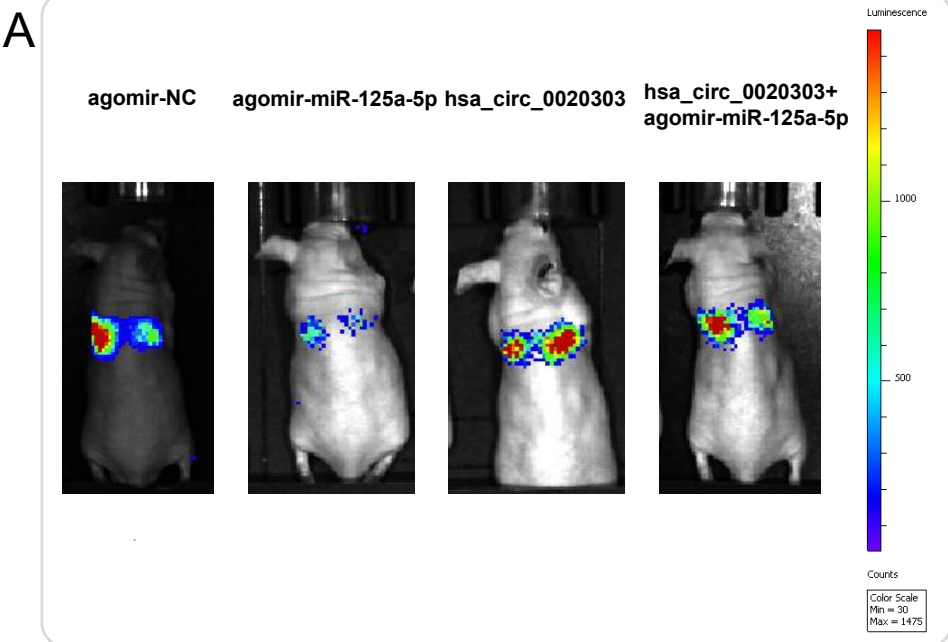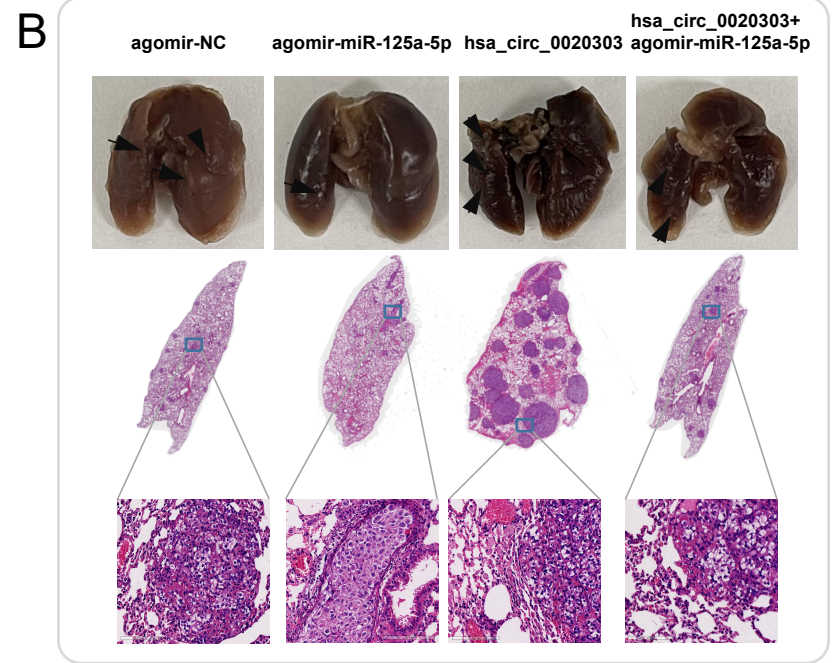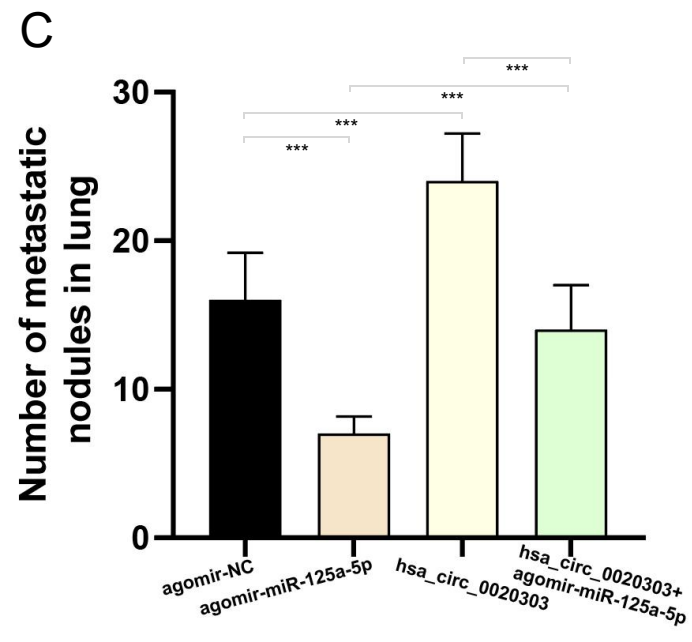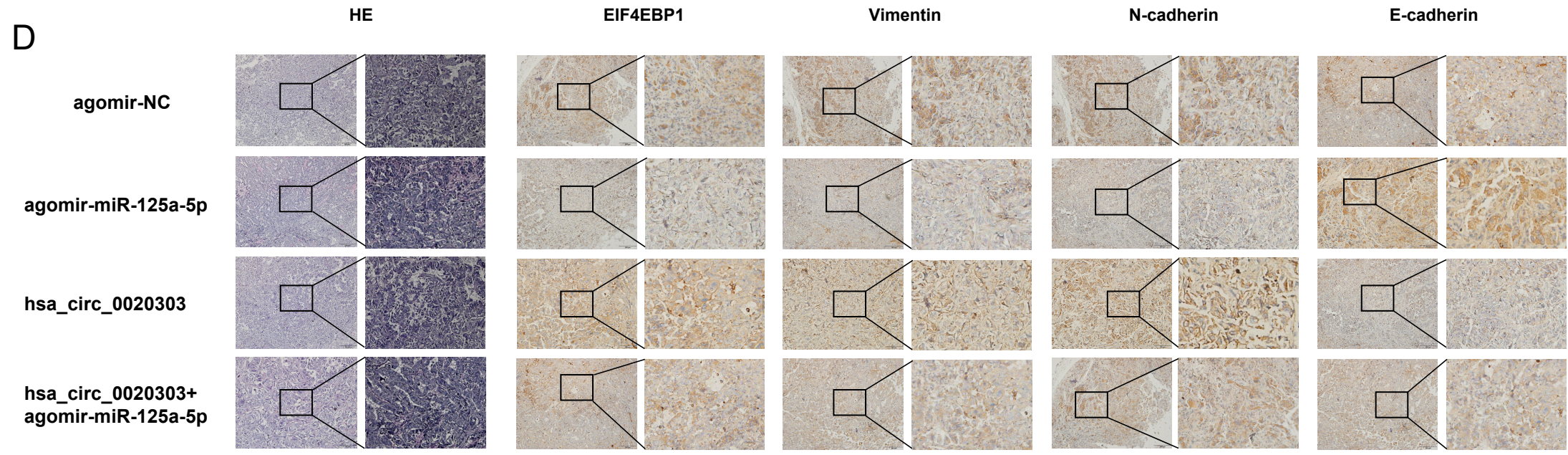

Supplement: Supplementary file 9 — Additional file 9: Figure S6. circCHST15 reverses the tumor-suppressing effect of miR-125a-5p on the metastasis of RCC cells in vivo. A. Bioluminescence of the lung metastatic nodules was detected by an in vivo bioluminescence imaging system. B. Representative images of HE staining analysis of the lung metastatic nodules in each group. C. lung metastatic nodules were enucleated and measured. The numbers of lung metastatic nodules were significantly decreased in agomir-miR-125a-5p group. However, circCHST15 reverses the tumor-suppressing effect of miR-125a-5p on the metastasis of 786-O cells in vivo. D. HE staining and IHC analysis of EIF4EBP1, Vimentin, N-cadherin and E-cadherin expression in subcutaneous xenograft tumors. circCHST15 reverses the tumor-suppressing effect of miR-125a-5p on the proteins’ expression of E-cadherin, EIF4EBP1, Vimentin and N-cadherin. [file 12943_2021_1449_MOESM9_ESM.pdf]
